# Molecular crosstalk between tumour and brain parenchyma instructs histopathological features in glioblastoma

**DOI:** 10.18632/oncotarget.7454

**Published:** 2016-03-25

**Authors:** Sébastien Bougnaud, Anna Golebiewska, Anaïs Oudin, Olivier Keunen, Patrick N. Harter, Lisa Mäder, Francisco Azuaje, Sabrina Fritah, Daniel Stieber, Tony Kaoma, Laurent Vallar, Nicolaas H.C. Brons, Thomas Daubon, Hrvoje Miletic, Terje Sundstrøm, Christel Herold-Mende, Michel Mittelbronn, Rolf Bjerkvig, Simone P. Niclou

**Affiliations:** ^1^ NORLUX Neuro-Oncology Laboratory, Department of Oncology, Luxembourg Institute of Health (L.I.H.) Luxembourg, Luxembourg; ^2^ Edinger-Institute (Neurological Institute), Goethe University, Frankfurt am Main, Germany; ^3^ Genomics and Proteomics Research Unit, Department of Oncology, Luxembourg Institute of Health (L.I.H.) Luxembourg, Luxembourg; ^4^ Core Facility Flow Cytometry, Department of Immunology, Luxembourg Institute of Health (L.I.H.) Luxembourg, Luxembourg; ^5^ U1029 INSERM, Angiogenesis and Cancer Microenvironment Laboratory, University of Bordeaux, Talence, France; ^6^ Division of Neurosurgical Research, Department of Neurosurgery, University of Heidelberg, Heidelberg, Germany; ^7^ NORLUX Neuro-Oncology, Department of Biomedicine, University of Bergen, Norway; ^8^ K.G. Jebsen Brain Tumour Research Centre, Department of Biomedicine, University of Bergen, Bergen, Norway; ^9^ Department of Pathology, Haukeland University Hospital, Bergen, Norway; ^10^ Department of Clinical Medicine K1, University of Bergen, Bergen, Norway; ^11^ Department of Neurosurgery, Haukeland University Hospital, Bergen, Norway

**Keywords:** glioblastoma, patient-derived xenograft, tumour microenvironment, endothelial cells, angiogenesis

## Abstract

The histopathological and molecular heterogeneity of glioblastomas represents a major obstacle for effective therapies. Glioblastomas do not develop autonomously, but evolve in a unique environment that adapts to the growing tumour mass and contributes to the malignancy of these neoplasms. Here, we show that patient-derived glioblastoma xenografts generated in the mouse brain from organotypic spheroids reproducibly give rise to three different histological phenotypes: (i) a highly invasive phenotype with an apparent normal brain vasculature, (ii) a highly angiogenic phenotype displaying microvascular proliferation and necrosis and (iii) an intermediate phenotype combining features of invasion and vessel abnormalities. These phenotypic differences were visible during early phases of tumour development suggesting an early instructive role of tumour cells on the brain parenchyma. Conversely, we found that tumour-instructed stromal cells differentially influenced tumour cell proliferation and migration *in vitro,* indicating a reciprocal crosstalk between neoplastic and non-neoplastic cells. We did not detect any transdifferentiation of tumour cells into endothelial cells. Cell type-specific transcriptomic analysis of tumour and endothelial cells revealed a strong phenotype-specific molecular conversion between the two cell types, suggesting co-evolution of tumour and endothelial cells. Integrative bioinformatic analysis confirmed the reciprocal crosstalk between tumour and microenvironment and suggested a key role for TGFβ1 and extracellular matrix proteins as major interaction modules that shape glioblastoma progression. These data provide novel insight into tumour-host interactions and identify novel stroma-specific targets that may play a role in combinatorial treatment strategies against glioblastoma.

## INTRODUCTION

Glioblastomas are still largely diagnosed based on neuropathological features including mitotic activity, extensive diffuse infiltration of central nervous system (CNS) tissue, pseudopalisading necrosis and microvascular proliferation, the latter correlating with blood brain barrier disruption [1]. It is not entirely clear to what extent histopathological characteristics are governed by inherent genetic properties of the tumour or are influenced by the local microenvironment. We and others have previously shown that patient-derived organotypic spheroid-based glioblastoma xenografts preserve key features of human glioblastoma in immunocompromised rats, including genomic abnormalities, extensive invasion into the brain parenchyma, neovascularization and comparable treatment responses, which are generally not observed in most transgenic and cell line-derived models [2-6]. Interestingly, in rats, xenografted patient-derived gliomas display tumour heterogeneity resulting in two major phenotypes: a highly invasive, angiogenesis-independent phenotype and an angiogenesis-dependent phenotype with moderate invasion. In some cases of prolonged serial passaging *in vivo*, the invasive tumours undergo an angiogenic switch suggesting progression to higher malignancy [3, 4]. We have previously shown that activation of the epidermal growth factor receptor (EGFR) is one of the mechanisms leading to the invasive, non-angiogenic tumour growth, while EGFRvIII expression appears to be associated with angiogenesis [7]. Thus, such models provide a means to dissect the principle features of glioblastoma in rodents and allow to interrogate the impact of tumour-host interactions on cancer growth.

Solid tumours can be considered as abnormal organs composed of neoplastic and non-neoplastic stromal cells embedded in a complex extracellular matrix (ECM) [8]. Each tumour type develops in a specific microenvironment and interactions between tumour and stromal cells are dynamic and influence tumour progression and drug resistance [9, 10]. Non-neoplastic cells contribute to the hallmarks of cancer, including sustained proliferation, angiogenesis, cell death and drug resistance [10, 11], therefore understanding the tumour-stroma crosstalk is crucial for the design of successful therapies. The brain parenchyma represents a unique niche for tumour development, encompassing glial cells, neurons, endothelial cells (ECs), pericytes, neural precursor cells, microglia and infiltrating immune cells such as tumour-associated macrophages, natural killer cells and T lymphocytes [12]. Here we established intracranial tumours in mice expressing eGFP, enabling a distinct analysis of the tumour and stromal compartments. At the microscopic level, we observed three distinct histological phenotypes in the mouse brain, for which we provide a detailed neuropathological and molecular characterization. We focus on the molecular interaction between tumour cells and ECs and provide evidence that pathophysiological features of glioblastoma are initiated by intrinsic genetic tumour characteristics and orchestrated by a reciprocal crosstalk between glioma and brain parenchyma, which reinforces the malignant phenotype.

## RESULTS

### Patient-derived glioblastoma xenografts give rise to three histological phenotypes

**Figure 1:**
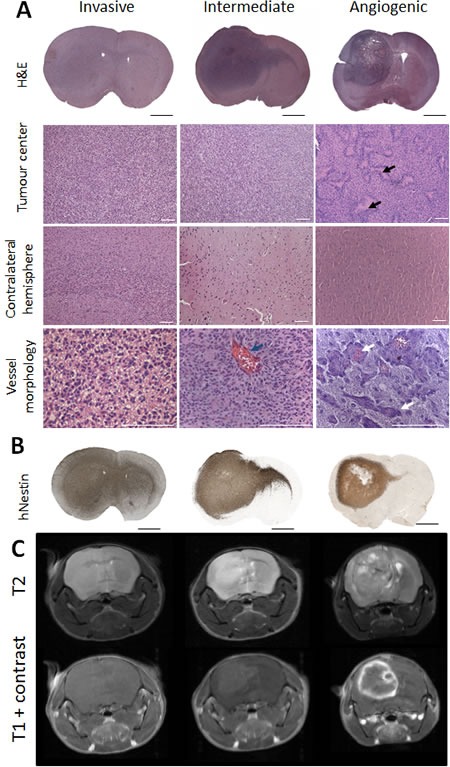
Phenotypic intertumoural heterogeneity in patient-derived glioblastoma xenografts Patient-derived tumour spheroids were implanted intracranially in NOD/Scid mice. Based on histological features, xenografts derived from different patients were classified into three groups: highly invasive, intermediate and angiogenic phenotypes. **A.** Hematoxylin/Eosin staining showing representative histology of the three phenotypic groups: invasive (P8), intermediate (P3) and angiogenic (P13). Tumour centre, contralateral hemisphere and blood vessel morphology are presented in higher magnification. Black arrows indicate areas of necrosis with pseudopalisading cells in the angiogenic tumour. Blue arrow highlights rare enlarged vessels in intermediate tumours. White arrows point to vessels with glomeruloid structures typical for angiogenic xenografts. Scale bars represent respectively 1 mm (black) and 100 μm (white). **B.** Presence of tumour cells in the tumour centre and varying levels of invasion to contralateral hemispheres in representative phenotypes were confirmed with human-specific nestin immunohistochemistry. **C.** T2 weighted MR images confirmed the presence of oedema in angiogenic and intermediate tumours (bright field at the tumour side). T1 weighted MRI scans with contrast agent showed the absence of contrast enhancement in invasive tumours, while intermediate and angiogenic tumours showed low and high contrast enhancement respectively (*n* = 4) (examples shown for P8 invasive, P3 intermediate and P13 angiogenic tumours). See Suppl. Table 1 and 2 for more information on patient material and PDX models.

Organotypic spheroids generated from twelve different glioblastoma patients (Suppl. Table 1) were derived from mechanically minced biopsies. After short-term culture (5-12days), spheroids from each patient were implanted into the brain of NOD/Scid mice. Intracranial tumours developed within 4 to 22 weeks depending on the parental tumour (Suppl. Table 2) and were further serially transplanted for up to seven generations. A total of 281 patient-derived xenografts (PDX) were analysed. Based on histopathological characteristics, all glioblastoma xenografts were classified into three major phenotypes: ‘invasive’, ‘intermediate’ and ‘angiogenic’ (Figure 1; Suppl. Table 2). The invasive phenotype (6/12) was characterized by a high density of tumour cells in the implanted and the contralateral hemisphere (Figure 1A-1B). These tumours displayed a very homogeneous morphology and were not easily distinguishable as autonomous tissue, since they were diffusely embedded in the residual brain parenchyma. Both hemispheres were almost entirely filled with invading tumour cells (Figure 1B). Five out of twelve glioblastomas gave rise to an intermediate and one to an angiogenic phenotype. The latter displayed a high density of tumour cells in the implanted hemisphere and only a limited number of cells invading *via* the corpus callosum (Figure 1A-1B). Angiogenic tumours presented areas with pseudopalisading necrosis (Figure 1A, black arrows), a large number of dilated blood vessels, often associated with prominent glomeruloid-like microvascular proliferations (Figure 1A, white arrows). Although most activated vessels still displayed a small lumen (Figure 1A lower right image), the ratio of lumen to vessel wall thickness was dramatically decreased suggesting a considerable impairment of vessel perfusion.

The intermediate phenotype presented less extreme features with a pronounced but intermediary invasion score. While in invasive tumours blood vessels displayed thin vessel walls and hardly any signs of endothelial cell activation, intermediate tumours occasionally displayed abnormal vessels however lacking microvascular proliferation (Figure 1A, blue arrow). Importantly, all spheroid-derived tumours grew diffusely with a certain level of invasion, contrary to the circumscribed tumours typically seen in adherent cell line-derived xenografts. For a given patient, the tumour take was very high (> 95%), the histological phenotype was reproducible and largely stable between different mice, including nude and NOD/Scid strains, and after serial transplantation.

Magnetic resonance imaging (MRI) confirmed the histological analysis, showing a brighter, homogeneous brain tumour area in T2 weighted images of the invasive phenotype (Figure 1C) and no contrast enhancement in T1 images, indicating an intact vasculature. In contrast, angiogenic tumours were heterogeneous in T2 images and displayed strong contrast enhancement, reflecting the pathological vessel morphology (Figure 1C). Intermediate tumours had a low contrast enhancement. Oedema was occasionally observed in intermediate and angiogenic tumours, recognized as hyperintense signal in FLAIR sequences within and around the tumour area (Suppl. Figure 1).

In summary, glioblastoma patient-derived xenografts based on organotypic spheroids can be reliably established in mice and maintain typical glioblastoma characteristics. Notably, depending on the parental tumour, they develop according to three distinct phenotypes that we classified as ‘invasive’, ‘intermediate’ and ‘angiogenic’. The separation of key glioblastoma features into different phenotypes provides an opportunity to study their development *in vivo* and raises the intriguing question as to why patient glioblastomas unfold into different phenotypes when transplanted into the rodent brain.

### Glioblastoma stem-like cells develop similar *in vivo* phenotypes as compared to patient-derived spheroids

To exclude that glioblastoma phenotypes are a result of the experimental procedure of spheroid generation, we analysed xenografts derived from five patient-derived glioblastoma stem-like cell lines. Interestingly, we observed three very similar phenotypes to those obtained with organotypic spheroids. Three cell lines (NCH601, NCH465 and NCH660h) gave rise to an invasive phenotype, one cell line (NCH421k) to an intermediate phenotype and one (NCH644) to a full-blown angiogenic phenotype with microvascular proliferation (Suppl. Figure 2A-2B). This suggests that the phenotypes are linked to inherent characteristics of the parental tumours, rather than to specific experimental procedures of sphere generation. The fact that stem-like cell lines provide similar *in vivo* phenotypes as PDX models, is also important in view of the fact that these are more amenable to genetic manipulation and *in vitro* studies.

### Phenotypic glioblastoma characteristics are present at an early stage of tumour development

**Figure 2:**
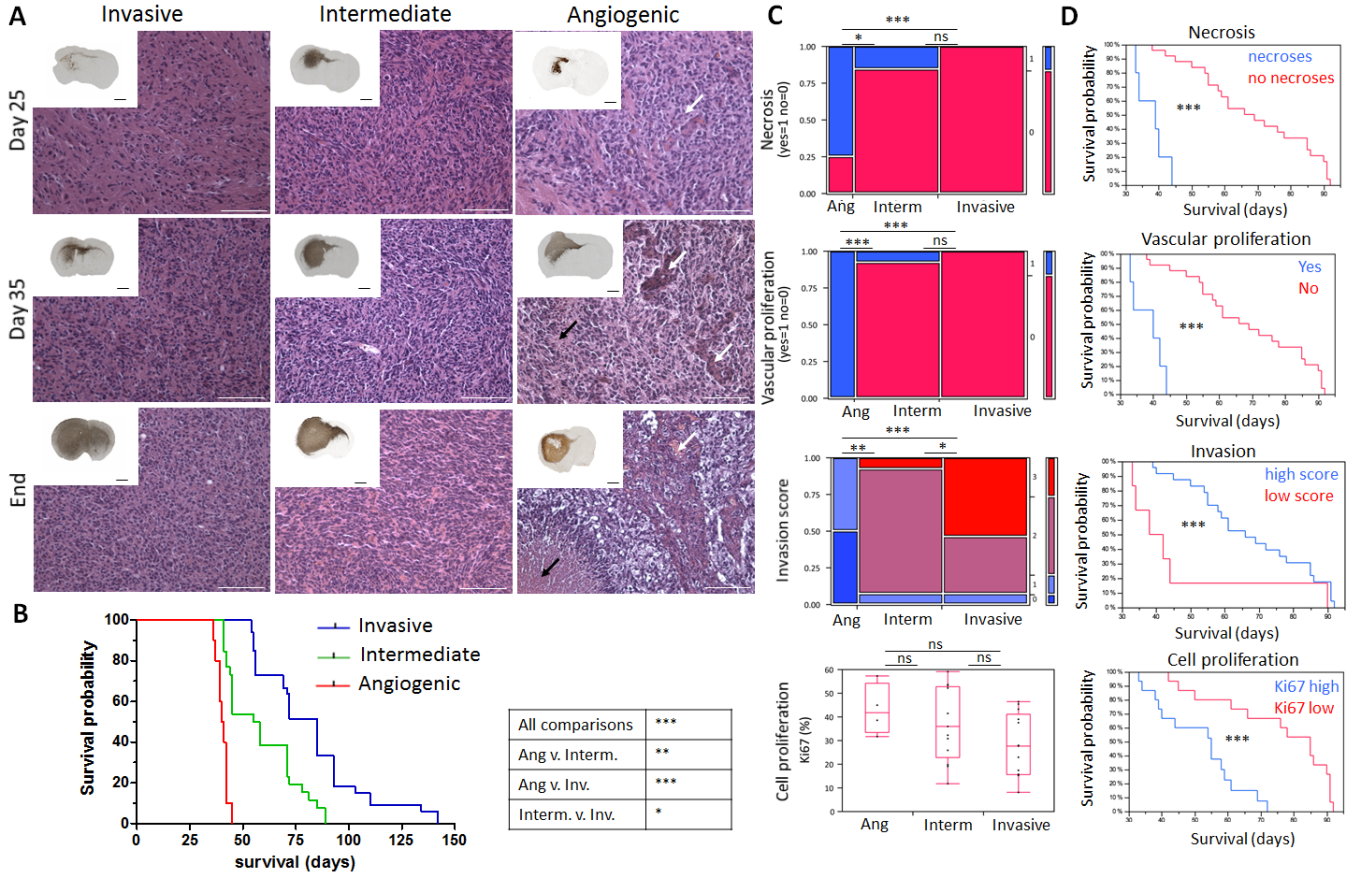
Time course of glioblastoma development and neuropathological analysis in patient-derived xenografts **A.** Tumours representing the three phenotypes were analysed at post-implantation day 25, 35 and at day of sacrifice (‘End’). Hematoxylin-Eosin (magnification) and human nestin-specific stainings (inserts) reveal clear histological differences between the phenotypes at all stages of tumour development. Black arrows represent areas of necrosis with pseudopalisading cells in angiogenic xenografts and white arrows show vessels with glomeruloid structures typically seen in angiogenic xenografts already at early time points. Black and white scale bars represent 1mm and 100 μm respectively (P8 invasive, P3 intermediate and P13 angiogenic, *n* = 2 per time point). **B.** Kaplan-Meier survival curves of xenotransplanted mice (generation 2) displaying invasive phenotypes (blue for P8, T101, T185, T233, T239, T251), intermediate phenotypes (green for P3, T16, T238, T341, T434) and angiogenic phenotype (red for P13). See Suppl. Table 2 for details on PDX models. **C.** Neuropathological analysis for necrosis (Yes/No), vascular proliferation (Yes/No), invasion (high/low invasion score) and cell proliferation (percentage of Ki67 positive cells) was performed for invasive (P8, T101, T185, T233, T239, T251), intermediate (P3, T16, T238, T341, T434, NCH421k) and angiogenic phenotypes (P13, NCH644); 30 mice in total. **D.** Kaplan-Meier survival curves of xenotransplanted mice based on the presence of neuropathological features; *p* values were calculated with the Wilcoxon signed-rank test; *p-value < 0.05, **p-value < 0.01, ***p-value < 0.001.

Tumour angiogenesis is normally expected to arise in solid tumours when a distinct size or distance from blood vessels has been reached [13, 14]. To assess the development of the phenotypes, we followed intracranial tumour development over time. Xenografted mice were analysed at 25 and 35 days post-implantation and at sacrifice (‘end’; Figure 2A). All animals displayed visible tumours at day 25 (Figure 2A, inserts). Notably, the typical features of each phenotype were already detectable at an early stage of tumour development. Invasion of tumour cells to the contralateral hemisphere was observed in all phenotypes, but was by far most prominent in the ‘invasive’ phenotype, with invasion into cortical structures. Abnormal vessels in angiogenic tumours were also already present at day 25 (Figure 2A, white arrows), suggesting that angiogenesis is an early event in tumour development and is not merely regulated by tumour size. The number of pathological vessels with microvascular proliferation further increased with time (day 35 and ‘end’), when also necrotic areas became apparent in the tumour core (Figure 2A, black arrows). Thus the typical glioblastoma features observed in the three phenotypes develop during early stages of tumour growth thereby presaging the final outcome of tumour histology.

### Tumour aggressiveness is linked to proliferation index, invasion, necrosis, and angiogenesis

To assess to what extent aggressiveness was associated with the tumour phenotypes, we correlated the mouse survival with the phenotype. We found that the angiogenic phenotype was most aggressive (mean survival 41.9+/−3.6 days, Figure 2B), while mice with intermediate tumours survived longer (mean survival 58+/−3 days) and mice with invasive tumours showed the longest survival times (mean survival 82+/−4 days). Mice with angiogenic tumours also died much faster after the onset of neurological symptoms, while mice with invasive tumours had less severe neurological symptoms and a more variable survival time. These observations were confirmed in the xenografts derived from stem-like glioblastoma cells, where the angiogenic tumours were also the most aggressive (Suppl. Figure 2C).

To quantify the observed histological differences we performed a detailed independent neuropathological analysis (Figure 2C). Necrosis and vascular proliferation were statistically different in angiogenic compared to invasive and intermediate phenotypes, whereas invasion was significantly different between the three phenotypes (Figure 2C). Although the proliferation index did not differ significantly between the phenotypes, there was a strong tendency for higher proliferative activity in angiogenic tumours (*p* = 0.06, Figure 2C). The presence of necrotic areas, angiogenesis, high proliferation (Ki67 index), and low invasion score were negatively correlated with survival (*p* ≤ 0.001) (Figure 2D). However multivariate analysis for independence of variables revealed that only the proliferation index was independent (*p* = 0.0001). This was exemplified by the observation that animals with invasive tumours could display a relatively high proliferation index and short survival, (*e.g.* P8), or a low proliferation index and long survival (*e.g.* T101) (Suppl. Table 2).

In summary, histopathological tumour phenotypes are correlated with mouse survival, with the angiogenic tumours being the most aggressive. Aggressiveness is correlated with high proliferation, microvascular proliferation, necrosis and low invasiveness, where only the proliferation index is independent of the phenotype.

### Distinct vessel morphology in histological glioblastoma phenotypes

**Figure 3:**
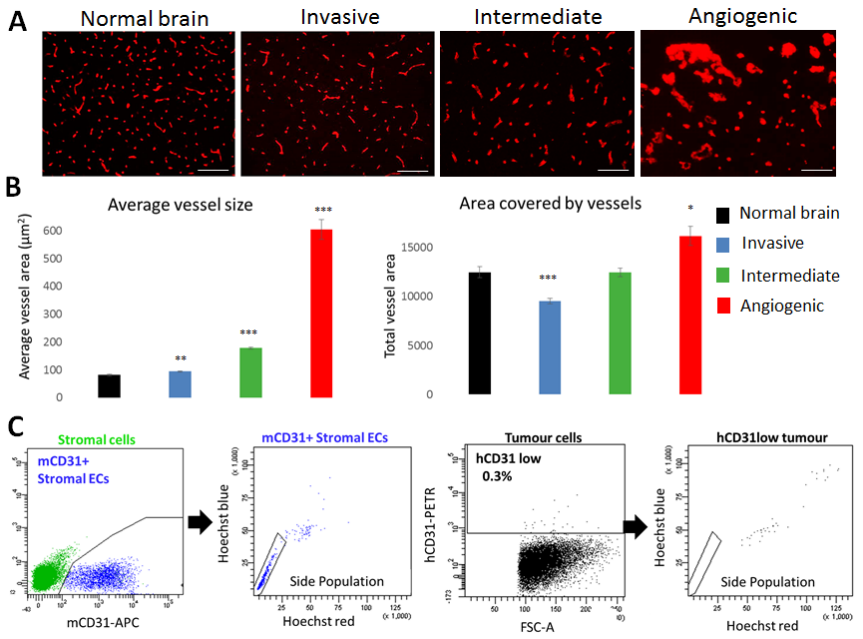
Vascular morphology in glioblastoma phenotypes **A.** Blood vessels from normal and tumour containing brains were visualized by mouse-specific anti-CD31 immunohistochemistry (Scale bars 100μm). Marked differences in blood vessel morphology were observed in the three tumour types (examples shown for P8 invasive, P3 intermediate and P13 angiogenic). **B.** Quantification of mCD31 staining to determine average vessel size and total vessel area. Results are displayed as mean +/− SEM. Xenografts used for quantification: (1) Invasive: T101, T185, T239, T251, P8; (2) Intermediate: P3, T16, T238, T434, T341, NCH421k; (3) Angiogenic: P13, NCH644 (*n* = 3 for each patient-derived xenograft; *p value < 0.05, **p value < 0.01, ***p value < 0.001). **C.** Functional flow cytometric analysis of CD31 positive cells in microenvironment (green, left) and tumour (black, right) compartments (examples shown for P3). Mouse CD31 positive ECs (blue) displayed the side population (SP) phenotype typical of brain endothelium. Rare human tumour cells with very low CD31 positivity were never in the SP. See Suppl. Figure 3 for more details on gating strategy.

In view of the differences in the vessel structure observed by histology and MRI, we performed a detailed analysis of the vessels in the three phenotypes. Immunostaining with a mouse-specific CD31 antibody confirmed a close to normal vessel morphology in invasive tumours, and a grossly aberrant morphology in the angiogenic phenotype (Figure 3A). Quantification of the average size per vessel revealed a 7.4-fold increase compared to normal brain, indicating major vessel abnormalities in the angiogenic phenotype (Figure 3B). A roughly 2-fold increase was seen for the intermediate phenotype, while vessels of the invasive phenotype were only slightly larger in size compared to normal brain. On the other hand, the overall vessel area was only barely increased in angiogenic tumours compared to normal brain, while it was decreased in invasive tumours (Figure 3B), most likely reflecting the ‘dilution’ of the vessel network in the tumour area. No change was seen for intermediate tumours.

It has previously been suggested that tumour stem cells contribute to vessel formation through a transdifferentiation process, although these data have been questioned by others [15]. By immunohistochemistry, we could not detect any human-specific CD31 staining in glioblastoma xenografts (not shown). Using flow cytometric analysis, we found that CD31 positive cells in the tumour were GFP-positive and thus host-derived, and displayed strong efflux properties (so-called ‘side population’ (SP) phenotype), a hallmark of brain ECs (Figure 3C, Suppl. Figure 3A-3B) [16]. Although by flow cytometry a CD31 signal was occasionally (0-0.4%) detected within tumour cells (black, Figure 3C), the signal intensity was close to background and was far from the CD31 expression level (up to 100 fold lower) typically seen in human ECs or HBMVEC cells (Suppl. Figure 3B-3C), and these cells did not exhibit efflux properties, indicating that they do not represent functional brain ECs (Figure 3C).

In conclusion, pathological tumour vessels are found in angiogenic and intermediate phenotypes, while invasive tumours retain a largely normal vessel morphology. Furthermore we show that ECs in glioblastoma are stroma-derived and do not detect any transdifferentiation of tumour cells into functional endothelium.

### Differentially instructed stromal cells modify tumour cell behaviour

**Figure 4:**
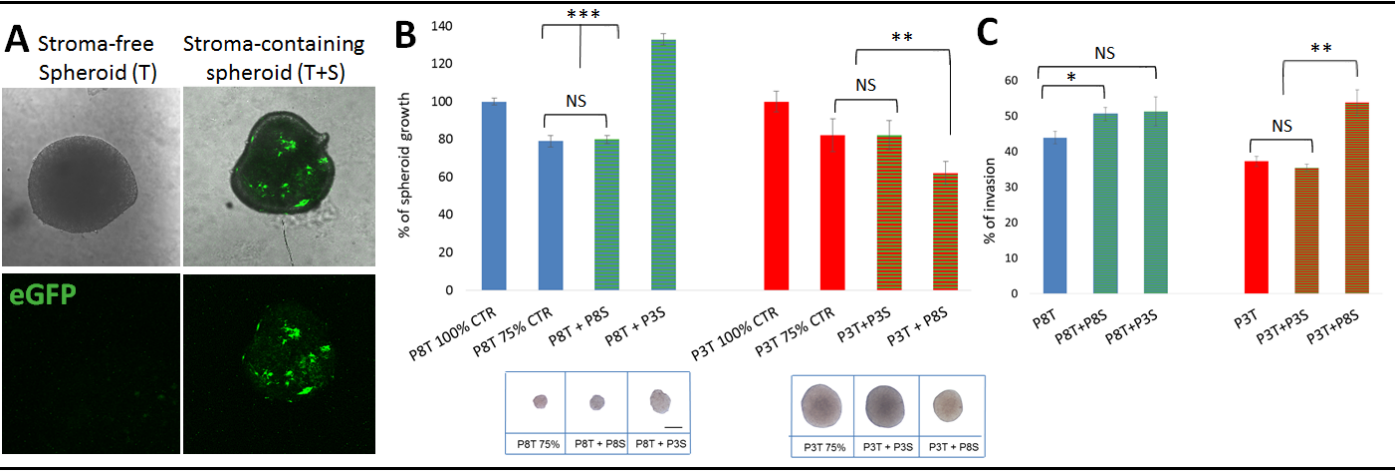
Tumour-instructed stromal cells affect tumour cell behaviour *in vitro* **A.** Sorted eGFP-negative tumour cells were plated in agar coated plates to form stroma-free spheroids (T). To obtain stroma-containing spheroids (T+S), sorted eGFP-negative tumour cells were premixed with sorted eGFP-positive stromal cells. **B.** Effect of stromal cells from different phenotypes on tumour spheroid growth. Stroma-containing spheroids were prepared with 500 stromal cells of the same tumour (P8T+P8S; P3T+ P3S; 25% of stromal cells) or with 500 stromal cells of another phenotype (P8T+ P3S; P3T+ P8S, 25% of stromal cells) pre-mixed with 1500 tumour cells. Two stroma-free control spheroids were considered: ‘100%’ control (2000 tumour cells) and ‘75%’ control (1500 tumour cells). Spheroids were cultured for 14 days and the spheroid area was determined at day 14 and 7. Results are presented as the percentage of the control spheroid (100%) of respective tumour cells. Images (insert below graphs) show representative spheroids after 14 days of culture. Scale bar represents 100μm; *p value < 0.05, **p value < 0.01, ***p value < 0.001 **C**. Tumour cells with or without stromal cells were plated in a Boyden chamber pre-coated with Matrigel and incubated for 96h; *p value < 0.05, **p value < 0.01).

The time course experiment suggested that tumour cells have an inherent capacity to modify and shape their microenvironment. Here we asked whether and how the glioma microenvironment is specifically ‘instructed’ to support tumour growth. Based on eGFP expression, we separated tumour and stromal cells by FACS analysis (Suppl. Figure 3A) and performed growth and invasion assays with either stroma-free tumour spheroids (T) or tumour spheroids re-admixed with stromal cells (T+S) (Figure 4A). Tumour growth was determined by measuring the diameter of spheroids at different time points and invasion was measured using a modified Boyden chamber assay [17]. Consistent with their *in vivo* growth, stromal-free spheroids of invasive tumours (P8T) showed little proliferation *in vitro* and remained similar in size, while spheroids of intermediate tumours (P3T) proliferated *in vitro* resulting in larger spheroids (Suppl. Figure 4A; Figure 4B, inserts). The addition of P8-derived stromal cells to P8 spheroids did not change their growth, but addition of P3-derived stromal cells strongly induced the growth of P8 spheroids (Figure 4B). Conversely, P8-derived stromal cells added to P3 spheroids inhibited growth, while stromal cells of the same origin had no effect. The basal invasion capacity of P8 tumour cells (‘invasive’) was higher than P3 tumour cells (‘intermediate’) (44% *vs* 37%, Figure 4C). Of note, the addition of P8 instructed stromal cells significantly increased the invasion of P8 tumour cells which was even more pronounced on P3 tumour cells (Figure 4C). Conversely P3 instructed stromal cells did not affect invasion in either case.

These data suggest that the brain parenchyma is differentially instructed by the tumour and reinforces the phenotypic tumour characteristics, indicating a reciprocal tumour-microenvironment interaction *in vivo*. Of note, the properties of stromal cells are retained *in vitro* and are likely the result of cell membrane or secreted factors enabling a direct crosstalk with tumour cells.

### Tumour-specific gene expression profiles reflect histological tumour phenotypes

We have previously shown that glioblastomas mostly comprise ECs, pericytes, microglia/macrophages and neural stem cells [16]. Because ECs constitute a major population of the glioma microenvironment and are the mediators of the angiogenic process, we focused on the interaction of tumour cells with ECs. Using eGFP-based FACS sorting (Suppl. Figure 3A), we specifically isolated ECs and tumour cells of the three phenotypes for transcriptomic analysis (P8 invasive, P3 intermediate, P13 angiogenic).

**Figure 5:**
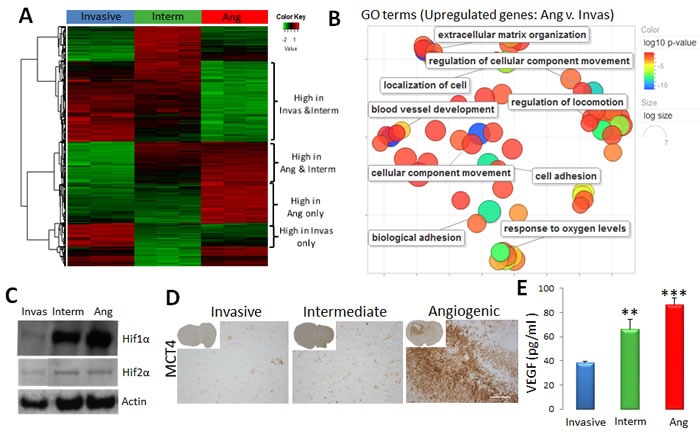
Tumour cell-specific gene expression profile of the three phenotypes **A.** Sorted tumour cells of invasive (P8), intermediate (P3) and angiogenic (P13) glioma xenografts were used for transcriptomic analysis (*n* = 3). Heatmap representing gene expression levels of differentially expressed genes between angiogenic, intermediate and invasive tumours (FDR < 0.01, abs(FC) > = 2). See Suppl. Table 4 for detailed gene lists. **B.** Revigo summary of main GO terms (DAVID^®^ database) up-regulated in angiogenic *versus* invasive tumour cells (FDR < 0.01, FC > = 2). Colour scale represents the log10 p-value; see Suppl. Table 5 for complete list of GO terms. **C.** Western Blot analysis showing increased levels of HIF1α and HIF2α proteins in angiogenic (Ang) and intermediate (Interm) tumours. Representative images were cropped from the same blots. **D.** Immunohistochemistry for MCT4 lactate transporter shows increased expression in the perinecrotic niches of angiogenic tumours. **E**. Elisa-based quantification revealed a gradual increase of VEGF in xenografts from invasive to angiogenic phenotype (mean +/− SEM; *n* = 3).

Our first analysis focused on the tumour-specific gene expression profiles. Using stringent analysis parameters, 2672, 2195 and 1724 differentially expressed genes (DEGs) between angiogenic vs. invasive; angiogenic vs. intermediate and intermediate vs. invasive tumour cells respectively were identified (Figure 5A). Cluster analysis confirmed that the intermediate phenotype shared molecular features with angiogenic and invasive tumours, thus representing a bonafide ‘intermediate’ phenotype also at the molecular level (Figure 5A, Suppl. Table 4).

Key pathways associated with the angiogenic tumour phenotype involved response to oxygen deprivation, vascular development and blood vessel remodelling, and extracellular structure organization, thus reflecting increased hypoxia and angiogenic processes in these tumours (Figure 5B, Suppl. Table 5). Many genes related to glycolysis such as *ALDOC*, *HK2*, *PGAM1* and *LDHA* were significantly up-regulated in angiogenic tumour cells (Suppl. Table 4). Similarly, angiogenesis-related genes, likely to impact neighbouring ECs, were significantly induced, including *VEGFA, TGFB1, ANXA2, NRP1, NRP2, MMP2, MMP14, PLAUR*, *NOS2* and *ANGPT2*. This was further corroborated by the upstream regulator analysis identifying the HIF1A transcription network to be activated in angiogenic tumours (Suppl. Table 6). Of note HIF2α (*EPAS1*) was also up-regulated at the transcriptional level in angiogenic and intermediate tumours (Suppl. Table 4). Increased hypoxia and glycolysis were further confirmed by the increased levels of HIF1α and HIF2α proteins in intermediate and angiogenic tumours (Figure 5C) and the presence of MCT4 in perinecrotic zones in the angiogenic phenotype (Figure 5D). Interestingly in culture, spheroids from all phenotypes expressed HIF1α (Suppl. Figure 4B), probably due to the presence of hypoxia in the spheroid core. The gradual increase of *VEGFA* from invasive to angiogenic tumours (Suppl. Table 4) was also confirmed at the protein level (Figure 5E).

Angiogenic tumours also differed with regard to expression of genes associated with cell motion, adhesion and extracellular matrix (Figure 5B; Suppl. Table 5). In particular we observed higher levels of extracellular matrix proteins such as *TNC*, *COL1A2*, *FN1* and ADAM-family proteases, as well as cytokines involved in immune cell recruitment including *TGFB1*, *IL7* and *CSF1* (Figure 5B, Suppl. Table 4). Pathways up-regulated in invasive tumour cells were associated with transcription regulation, neuronal development and cell morphogenesis (Suppl. Table 5).

In conclusion, differential gene expression profiles of the tumour cell compartment reflect the three distinct histological glioblastoma phenotypes observed in xenografts. The data suggest that tumour cells of the angiogenic phenotype display a hypoxia-driven gene expression programme and release a variety of factors that may shape the surrounding microenvironment.

### Endothelial cells undergo phenotypic adaptation in response to angiogenic tumours

**Figure 6:**
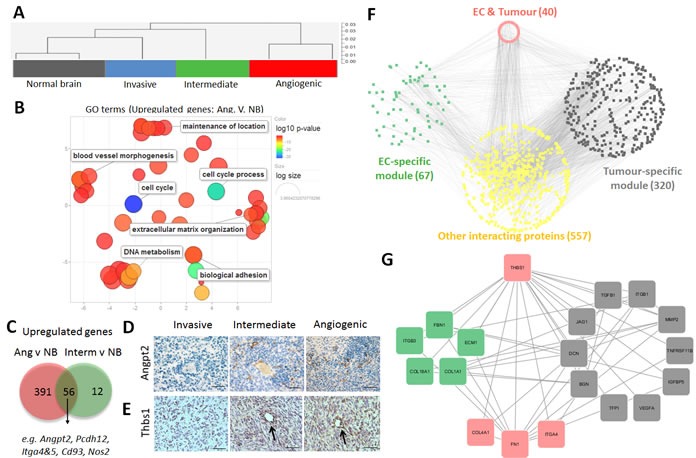
Phenotypic adaptation of endothelial cells in angiogenic tumours **A.** Sorted ECs (eGFP^+^CD31^+^) of normal brain (NB), invasive (P8), intermediate (P3) and angiogenic (P13) glioblastoma xenografts were used for gene expression analysis. Cluster analysis of gene expression profiles indicates that xenograft-derived ECs are more closely related to each other and differ from normal brain ECs. **B.** Revigo summary of main GO terms (DAVID^®^ database) up-regulated in ECs within angiogenic tumours compared to normal brain (FDR < 0.01, any fold change). Color scale represents the log10 p-value. See Suppl. Table 7 for complete list of GO terms. **C.** Venn diagram showing the comparison of up-regulated DEGs (see Suppl. Table 8 for detailed list of DEGs). **D.**-**E.**. Immunohistochemistry confirming increased expression of Angiopoietin 2 (Angpt2), D and Thrombospondin 1 (Thbs1), E in ECs of the intermediate and angiogenic tumours (D: scale bar 30μm; E: scale bar 20μm). Note that increased expression is also visible in the tumour cells of the angiogenic phenotype. **F.** Integrative protein-protein interaction analysis using genes up-regulated in tumour cells and ECs of the angiogenic phenotype (selected tumour list: cell membrane and extracellular matrix proteins, P13*vs*P8 cells, FDR < 0.01, FC > = 2; selected EC list: cell membrane and extracellular matrix proteins P13*vs*NB, FDR < 0.01, FC > 1;). The network shows direct protein-to-protein interactions between tumour-specific modules (‘grey’) and EC-specific modules (‘green’), as well as indirect interactions *via* putative protein partners (‘yellow’). Genes expressed by both modules are indicated in the ‘red’ module. Number of genes per module is displayed in brackets. **G.** Selected network of protein-protein interactions for THBS1. THBS1 and its first neighbours are displayed in a circular layout grouped by category (green: expressed in ECs; grey: expressed in tumour; red: expressed in both cell types). Only the direct interactions between molecules up-regulated in tumor and ECs are shown. (THBS1: thrombospondin-1, FBN1: fibrillin-1, ECM1: extracellular matrix protein-1, COL1A1: collagen-1a1, COL1BA1: collagen-1ba1, ITGB3: integrin-b3, TGFB1: transforming growth factor B1, ITGB1: integrin-b1, MMP2: matrix metalloproteinase-2, TNFRSF11B: tumour necrosis factor receptor superfamily, member 11b, IGFBP5: insulin-like growth factor binding protein 5, VEGFA: vascular endothelial growth factor 1, TFP1 : transferrin pseudogene 1, BGN : biglycan, DCN : decorin, JAG1 : jagged 1, ITGA4 : integrin-a4, FN1 : finbronectin-1, COL4A1 : collagen-4a1).

Gene expression profiles were interrogated in ECs isolated from the three tumour phenotypes. As control we used ECs isolated from normal brain (NB) of NOD/Scid mice. Cluster analysis showed that ECs from tumours displayed genome-wide transcriptomic changes compared to normal brain ECs (Figure 6A). The most pronounced changes were observed in ECs from angiogenic tumours (635 DEGs for P13 vs.NB), where functional pathway analysis pointed to the induction of cell cycle and proliferation activity, induction of vascular development and angiogenesis (*e.g. Mmp14, Col1a1, Tnc, Nos2, Angpt2, Angpl4, Thbs1, Tnfaip2*) (Figure 6B, Suppl. Tables 7, 8). Similarly a large number of genes coding for extracellular matrix and adhesion proteins were up-regulated: collagens (*Col4a2, Col6a3, Col18a1, and Col15a1*), proteases (*Adamts2, Adamts7 Adamts8, Adamts12, Adam12),* integrins (*Itga2, Itga3, Itga4, Itga5, Itgb3*), laminins *(Lama4, Lamc1), Fn1, Hspg2*, *CD93* and *Bmp1* (Figure 6B, Suppl. Table 8). Genes downregulated in ECs were associated with membrane organisation, cell projection, ion homeostasis and transport (Suppl. Table 7).

Most of the up-regulated genes were common in ECs of intermediate and angiogenic phenotypes, although ECs of the angiogenic phenotype appeared more activated with higher number of DEGs (Figure 6C). For example, *Angpt2* which was up-regulated in intermediate and angiogenic ECs (Suppl. Table 8) was confirmed at the protein level in both phenotypes (Figure 6D). Interestingly the increase of *ANGPT2* observed at the tumour cell level (Suppl. Table 4) was also confirmed by IHC (Figure 6D). Of note Angpt2 protein expression in blood vessels was correlated with tumour aggressiveness (Suppl. Figure 5). Expression of the TGFβ activator *Thbs1* which was elevated in angiogenic ECs (Suppl. Table 8), but not in ECs of normal brain, was also confirmed by immunostaining (Figure 5E). ECs of intermediate tumours showed weak positivity for Thbs1. The immunostaining confirmed also increased levels of *THBS1* in angiogenic and intermediate tumor cells (Suppl. Table 4).

‘Upstream regulator analysis’ proposed the transcription factors Forkhead box protein M1 (FOXM1), a regulator of the cell cycle, and FOXO1, responsible for gluconeogenesis and angiogenesis, as the most prominent regulators of EC activation (Suppl. Table 9) whereas Tp53, a negative regulator of the cell cycle, was inhibited in angiogenic ECs. HIF1A network was also activated, suggesting an adaptation to the hypoxic tumour environment. Interestingly, several activated upstream regulators (HIF1A, TGFB1, TNF, ERK ERBB2) were common in angiogenic tumour cells and angiogenic ECs, suggesting that angiogenesis may be regulated by concerted regulatory gene expression networks from the two cell types (Suppl. Tables 6, 9).

### Crosstalk between tumour and endothelial cells

In addition to the common upstream regulators, we identified 75 genes that were concomitantly up-regulated in tumour cells and ECs of the angiogenic phenotype (Suppl. Table 10). These included genes for extracellular matrix proteins (*FN1, NID1, NID2, LAMB1, LAMC1, LGALS1, TNC*) and their modulators (*ADAM12, ADAM19, MMP14, PLAUR, SERPINE1, THBS1*), cell adhesion molecules/cell surface receptors (*CD44, ITGA4, KCNJ2, IGF2R, NRP2, PLXND1)* and ligands (*BMP6, CTGF*) (Suppl. Table 10 and 11). Many genes were also related to angiogenesis such as *ANGPT2*, *ANGPTL4*, *MMP14, ADAM12, TNC, THBS1* and *FN1*.

In an effort to identify the key molecular determinants of the reciprocal crosstalk between angiogenic tumour and ECs, we interrogated putative interactions between proteins encoded by upregulated genes. This was done by computationally integrating multiple publicly-available protein-protein interaction (PPI) databases. We focused on tumour DEGs *versus* EC genes up-regulated in the angiogenic phenotype and representing cell membrane, extracellular matrix and secreted molecules (resulting in 1004 proteins and 3240 PPIs) (Figure 6F). This analysis indicates direct PPIs between two compartments, but also indirect connections *via* other proteins not encoded by the DEGs, and offers a systems-level characterization of the molecular cross-talk between these two cell types. It underscores that such an interplay is highly inter-connected and can be mediated by multiple functionally-related proteins.

By further focussing on the direct connections between the two cell types, we identified numerous interactions within extracellular matrix and plasma membrane proteins associated with angiogenesis (Suppl. Figure 6A, Suppl. Table 11). Several molecules appeared to interact with a large number of partners from both compartments (Suppl. Figure 6A). This included EC-specific proteins (e.g. COL1A1, FBN1), tumour-specific proteins (e.g. ITGB1) and proteins up-regulated in both cell types (FN1, THSB1, CD44, ITGA4, CTGF). Notably, we identified a potential bi-directional crosstalk encompassing TGFB1, THBS1, integrins, FN1 and other key extracellular matrix molecules (collagens, growth factors and proteases) (Figure 6G and Suppl. Figure 6). Of note, while TGFB1, a master regulator of the malignant phenotype, appeared to be mostly up-regulated in the tumour, its activators THBS1, FN1 and ITGA4 were strongly induced in tumor cells and ECs, highlighting a concerted molecular interaction to regulate cellular activity.

In summary, we show that through autocrine and paracrine interactions tumour and ECs cooperate to create a unique angiogenic, tumour promoting microenvironment and we identify key molecules that represent attractive targets to modulate essential steps of this tumour-microenvironment crosstalk.

## DISCUSSION

Tumours leverage their surroundings to progress and evade therapy. Thus, a better understanding of the interactions between tumour and microenvironment may improve our ability to provide effective treatment. Here, we provide a detailed histological and molecular characterization of glioblastomas and their surrounding brain parenchyma, with an emphasis on the crosstalk between tumour and endothelial cells. We used human-to-mouse patient-derived glioblastoma xenografts, which similar to our previously described rat models [4, 6], were generally reproducible and stable over time. Notably, the PDX model reflected tumour heterogeneity and distinguished three major histopathological phenotypes: [1] a highly ‘invasive’ phenotype, [2] an ‘angiogenic’ phenotype and [3] an ‘intermediate’ phenotype. The latter phenotype may be considered as the most clinically-relevant model displaying all the typical features of glioblastoma, while the other two enable a separate interrogation of the angiogenic and/or invasive component of glioblastoma in patient-derived pre-clinical models. We found that angiogenesis was associated with increased tumour malignancy and decreased mouse survival, in line with clinical data (progression from low to high grade gliomas) and with numerous experimental studies [18-20]. The more pronounced neurological symptoms are likely due to the presence of oedema and strong intracranial pressure.

The origin of the three phenotypes is currently not fully understood. It should be noted that the here described histological features are present at variable intensities in all human glioblastomas (hence the diagnosis), sometimes juxtaposed in distinct patterns within the same tumour (intra-tumour heterogeneity). The PDX model may therefore reflect the spatial differences of the original tumour. Although we cannot rule out this hypothesis, it should be noted that all biopsies were derived from the contrast-enhancing tumour core and their spatial origin can thus not simply be correlated to the PDX phenotype. Alternatively, the phenotype may result from the patient-specific genetic profile of the tumour (inter-tumour heterogeneity), which induces specific changes to its microenvironment. In agreement with this, the characteristics of each phenotype were detectable at early stages of tumour growth. We have recently shown in the rat model that invasive tumour growth is strongly associated with EGFR gene amplification and receptor activation [7]. This tendency was confirmed in the current study (Suppl. Table 1), however not all invasive tumours showed EGFR amplification, implying that additional factors are involved.

A limitation of current PDX models for the study of tumour-host interactions is the requirement of immunodeficient animals. In this regard it is worthy to note that nude mouse strains still carry active microglia/macrophage populations, which are the main immune component in the brain. Of note we did not observe any difference between xenografts generated in nude or NOD/Scid mice, while macrophages are largely defective in the latter.

Using cell type-specific transcriptomic analyses, we find that the histopathological features are clearly reflected at the molecular level. Tumour cells of the angiogenic phenotype displayed higher levels of hypoxic (HIF1α, HIF2α, MCT4) and angiogenic markers (VEGF, ANGPT2). Of note, ANGPT2, generally thought to be only expressed in ECs, was also up-regulated in tumour cells. As pathological vessels were observed early during tumor development, vascular modifications seems to develop before the onset of hypoxia, possibly as a result of genetic determinants of the tumour leading to a pseudo-hypoxic state e.g. a constitutively activated HIF1α pathway in non-hypoxic conditions could induce angiogenesis, although other genetic factors may be involved. Collectively these data suggest that tumour angiogenesis is likely the combined result of genetic determinants of the tumour and the response to specific microenvironmental conditions. We have previously shown that ‘intermediate’ (P3) and ‘angiogenic’ (P13) PDX tumors adapt to anti-angiogenic treatment by switching to a glycolytic metabolism and increasing invasion [6, 21]. Interestingly, the morphological response to bevacizumab was more pronounced in tumors with a high level of angiogenesis [22], and our preliminary data indicate that this may also be true with regard to survival (unpublished), suggesting that the level of angiogenesis (and presence of angiogenic markers) may indicate whether patients could benefit from anti-angiogenic treatment.

eGFP expression in the host microenvironment allowed us to unequivocally distinguish host endothelial cells from neoplastic human cells. Based on combined GFP/CD31 staining and functional EC properties (SP phenotype), we could not find any evidence for transdifferentiation of tumor cells into endothelium indicating that blood vessels in glioblastoma originate from the stroma. This does not exclude the existence of vascular mimicry, where tumor cells without EC properties align to form blood cell-containing vascular channels [23], which we occasionally observe in glioma xenografts [24].

ECs from all three glioblastoma phenotypes displayed a different gene expression profile compared to normal brain endothelium. This indicates an early onset of molecular alterations in the vasculature even in the invasive compartment, where at the morphological level the vessels were indistinguishable from normal vessels. ECs from angiogenic tumours showed the most drastic adaptation and displayed altered biological functions similar to the tumour cells themselves, e.g. extracellular matrix organization, blood vessel morphogenesis, biological adhesion. Our data are in line with a recent elegant study profiling the transcriptome of human vessels in glioblastoma patients, although that study was limited by the fact that ECs could not be reliably separated from pericytes or other stromal cells [25]. Nonetheless a significant number of genes were also identified in the present EC-specific profile, e.g. *ANGPT2, CD93, FN1, ITGA5, NID1, NID2* and G2 checkpoint kinase: *WEE1* (Suppl. Table 12 full list of common genes). This does not exclude that pericytes, known as a major component of the aberrant tumour vasculature [26], may also express these genes. Of note, we find that WEE1, which was proposed as an anti-tumour target in glioblastoma and whose expression negatively correlates with patient survival [27], is up-regulated in angiogenic ECs. Targeting WEE1 may thus also affect tumour angiogenesis.

In line with a co-evolutionary model of neoplastic and non-neoplastic stromal cells, we identified many genes that were concomitantly induced in ECs and tumour cells in the angiogenic phenotype, and some of these showed the highest number of putative protein to protein interactions (*FN1, THBS1, CD44, ITGA4, CTGF*). FN1, a downstream target and regulator of TGFB1, which plays a key role in matrix assembly [28] was recently associated with glioma cell cohesion and motility, as well as tumour angiogenesis [29]. While TGFB1 was induced in the tumour, its activators integrins and THBS1 were upregulated in both cell types, highlighting the reciprocal crosstalk between the two cell types to regulate cell adaptation and tissue remodeling. This reciprocity of tumour-microenvironment interactions was also confirmed *in vitro*: activated stromal cells retained their glioblastoma specific phenotype and were able to influence tumour cell properties *in vitro*. In future studies it will be interesting to address the role of inter-cellular exchange of genetic and/or protein material between tumour and stromal cells *via* vesicles or tunneling nanotubes as has recently been reported for other cancers [30, 31].

Like VEGFA, TGFB1 was gradually increased from invasive to angiogenic tumor cells (Suppl. Table 4) and was also identified as a potential upstream regulator in both tumour cells and ECs, suggesting that this cytokine is a master regulator of tumour-microenvironment interactions in angiogenic tumours. Thus TGFB1 may be a promising target for treatment in combination with anti-VEGF targeting agents. It should be noted that TGFB1 has a dual role in regulating invasion through induction of the process of epithelial to mesenchymal transition (EMT) [32, 33], but in the presence of VEGF, these two growth factors cooperate to regulate angiogenesis [34]. Several anti-TGFβ strategies are evaluated in clinical trials, including anti-sense oligonucleotides (Trabedersen) and small molecule inhibitors (galunisertib), but to our knowledge no combination therapies with anti-angiogenic agents have so far been reported [35, 36].

Taken together, we provide a detailed histopathological and cell type-specific molecular characterization of glioblastoma in orthotopic PDX models. Our data highlights the intricate reciprocal crosstalk between tumour and brain parenchyma and identifies key molecular players that govern tumour-host interactions. The combined targeting of tumour-specific oncogenic pathways and key microenvironmental modulating pathways may deliver more efficient therapeutic strategies against glioblastoma.

## MATERIALS AND METHODS

### Clinical samples and animal studies

Glioblastoma samples were collected at the Centre Hospitalier in Luxembourg (Neurosurgical Department) and the Haukeland University Hospital (Bergen, Norway) from patients who gave informed consent. All studies were conducted according to the Declaration of Helsinki, and with approval from the local ethics committees (National Ethics Committee for Research (CNER) Luxembourg, local ethics committee Haukeland University Hospital, Bergen). All tumorectomies were from grade IV glioblastomas (WHO grading system, detailed patient information in Suppl. Table 1). One sample was available per patient, which was collected from the contrast-enhancing tumor zone.

From the available tumor tissue a small piece was flash-frozen for further molecular analysis, the remaining was mechanically minced without enzymatic digestion for generation of organotypic spheroids as previously described [16]. Minced tumour pieces were seeded on agar coated flasks, forming spheroids within 7-10 days in DMEM, 10% FBS, 2mM L-Glutamine, 0.4mM NEAA and 100U/ml Pen-Strep (all from Lonza). Organotypic spheroids were not further cultured, but either frozen for storage or immediately implanted into the right frontal cortex of NOD/SCID mice (6/mouse, diameter 300-400μm). Where indicated mice expressing enhanced green fluorescent protein (eGFP) were used [24]. A total of 281 mice were implanted with patient-derived spheroids and 59 mice with stem-like cell lines (50′000 cells /mouse). Mice were maintained under SPF conditions and were sacrificed upon neurological (locomotor problems, uncontrolled movements) or behavioral abnormalities (prostration, hyperactivity). Where indicated, first generation xenografts were serially implanted for several generations. The handling of animals was performed in accordance with the Luxembourgish law (based on the European Directive 2010/63/EU) and the Norwegian Animal Act. The local authorities and ethical committees for animal welfare approved the protocols (detailed xenograft description in Suppl. Table 2).

### Cell cultures

Five glioblastoma stem-like cell lines (NCH421k, NCH465, NCH601, NCH660h and NCH644) were generated from glioblastoma patient material [37] by enzymatic dissociation of the tumor tissue and culture of a single-cell suspension in serum-free medium (DMEM-F12 medium (Lonza) containing 1xBIT100 (Provitro), 2mM L-Glutamine, 30U/ml Pen-Step, 1U/ml Heparin (Sigma), 20ng/ml bFGF (Miltenyi) and 20ng/ml EGF (Provitro)). Such cell lines grow and are maintained as non-adherent spheres. Human brain microvascular endothelial cells (HBMVECs, kindly provided by Prof. Thomas Würdinger [38] were cultured in EC medium (EBM-2/EGM*^®^*-2-BulletKit*^®^*; LONZA) on fibronectin pre-coated surface.

### Magnetic resonance imaging

MR images were acquired on a 7T Pharmascan small animal MR scanner (Bruker Biospin GmbH) with a quadratic mouse head transmitter/receiver coil. Animals were anesthetized with 1-2% isoflurane mixed with 50% air and 50% O_2_, and placed lying prone in a cradle equipped with a heating pad set to 37°C. MRI sequences used included T2-weighted (Fast Spin Echo, TE/TR:36ms/4.3s), and T1-weighted (Fast Spin Echo, TE/TR: 9ms/1s) after injection of contrast agent (0.1 mL Gadodiamide, Omniscan, GE-Healthcare 0.5mmol/mL injected subcutaneously 5min prior to the scan). In plane resolution was 0.078mm * 0.078mm and slice thickness 1mm.

### Immunohistochemistry

Coronal sections (8μm) from paraffin-embedded brains were stained with hematoxylin (Dako) and 1% eosin (Sigma). For immunostaining, sections were pre-treated for 5min with Proteinase K (Dako) followed by 30min incubation at 95°C in retrieval solution (Dako). The Dako Envision+System-HRP was used following the manufacturer's instructions. Primary and secondary antibodies were incubated for 1h. Signal was developed with 3,3′-diaminobenzidine chromogen in 5-20 min. IHC of mouse endothelial cells was performed on isopentane flash-frozen tissues and cryostat sections (10μm) were fixed with acetone and chloroform. Nonspecific binding was blocked with 2% FBS in TBS and antibodies were incubated for 1 hr at RT. Pictures were acquired with a Leica DMI 6000B microscope. Vessel quantification was done using ImageJ software. Additional IHC preparations were performed using a Discovery XT automated staining module (Ventana) and standard protocols (list of antibodies in Suppl. Table 3).

### Neuropathological analysis

Neuropathological analysis and quantification was performed independently by two experienced neuropathologists (PNH, MM). The existence of necrosis (yes *vs*. no), vascular proliferations (yes *vs*. no) and the degree of invasiveness was assessed on the basis of hematoxylin-eosin (H&E) stained sections (invasion score; 0: mild diffuse invasion, rather sharply delineated tumour bulk; 1: mild diffuse invasion, rather sharply delineated tumour bulk with mild invasion of the corpus callosum; 2: moderate to strong diffuse infiltration of one brain hemisphere; 3: massive diffuse infiltration of both hemispheres, gliomatosis-like pattern). Additionally, we analysed the proliferative capacity (Ki67 positive cells / whole cell population (in %)) and Angpt2-positive vessels per mm^2^. A significance level was set for *p* < 0.05. Kaplan-Meier survival analyses were performed after median split and dichotomized in high or low (for Angpt2 and Ki67) or dichotomized for: necrosis: yes *vs*. no; vascular proliferations: yes *vs*. no; invasion: high > 1 *vs*. low < 1. All statistical analyses were performed using JMP 8 and JMP 11 (SAS, Cary, NC, USA). Kaplan-Meier survival analyses including Log-Rank and Wilcoxon testings were performed to assess the influence of neuropathological features on mice survival. Pearson's and likelihood ratio chi-squared tests were performed to analyze neuropathological differences between the groups. Multivariate analyses for independence were performed with the Ch2 test including the following parameters: necrosis, vascular proliferation, invasion score, proliferation index. Other analyses were performed with the two-tail Student's *t*-test. Significance was set for: *p-value < 0.05, **p-value < 0.01, ***p-value < 0.001.

### Spheroid growth assays

Spheroids free of stromal cells were obtained from sorted eGFP-negative tumour cells by plating 2000 (‘100% control’) or 1500 (‘75% control’) cells per well of 96 well plates pre-coated with agar. To obtain stroma-containing spheroids 1500 sorted tumour cells (75%) were premixed with 500 sorted eGFP-positive stromal cells (25%). The growth of spheroids was monitored during a 2-week period and pictures (Leica DMI 6000B microscope) were taken after 1, 7 and 14 days of culture. Spheroid area was calculated with Image J and the growth ratio was obtained by comparing the spheroid areas at day14 to day 7.

### Boyden chamber invasion assay

Invasion assays were performed in Boyden chambers as previously described [17] using thincerts (8μm pore size, Greiner Bio One) pre-coated with Matrigel^®^ (BD Biosciences). Sorted eGFP-negative tumour cells were used alone (5×10^4^ cells/well) or premixed with eGFP-positive stromal cells (5000 cells/well = 10% of tumour) before plating. After 96 hours the cells on both sides of the membrane were determined by cell labelling (Vibrant^®^ MTT Cell Kit Assay, Molecular Probes). Cell invasion was normalised to the total number of cells, thus correcting for cell proliferation (*n* = 2; from at least 3 animals each).

### Cell membrane phenotyping and sorting

Glioblastoma xenografts and control mouse brains were dissociated with the MACS Neural Tissue Dissociation Kit (P) (Miltenyi) following the manufacturer's instructions. For phenotyping flow experiments were performed as described [16]. Single cell suspensions were resuspended in DMEM, containing 2% FBS, 10mM HEPES pH 7.4 and DNAse I (10μg/ml; Sigma) at 1×10^6^ cells/ml followed by 90min incubation with Hoechst 33342 (5μg/ml, Bisbenzimide, Ho342; Sigma) at 37°C. After washing, cells were resuspended in ice-cold HBSS 2% FBS, 10mM HEPES pH 7.4 buffer (100 μl/test). Prior to flow cytometric analysis, cells were incubated with the IR-LIVE/DEAD^®^ Fixable Dead Cell Stains (Invitrogen; 1μg/ml) and appropriate preconjugated antibodies for 30 min at 4°C in the dark (Suppl. Table 3). Data acquisition was performed on a FACS Aria^TM^ SORP cytometer (BD Biosciences) fitted with a 632nm (30mW) red laser, a 355 (60mW) UV laser, a 405nm (50mW) violet laser and a 488nm (100mW) blue laser. For cell sorting, single cell suspensions were stained with the IR-LIVE/DEAD^®^ Fixable Dead Cell Stain and the mouse-specific CD31 antibody for 30 min at 4°C. eGFP-negative tumour cells were sorted to the cold flow cytometry buffer and used directly for RNA extraction with the QIAGEN^®^ RNeasy Mini Kit (Qiagen) (*n* = 3). eGFP^+^CD31^+^ mouse endothelial cells from xenografts and control mouse brains were sorted directly to Trizol^®^ LS (Life Technologies) (*n* = 2-3; from at least 3 animals each).

### Genome-wide gene expression analysis

Total RNA was extracted using the QIAGEN^®^ RNeasy Mini Kit (human-specific microarrays) or the Trizol^®^ LS extraction kit (Life Technologies) (mouse-specific microarrays), according to the manufacturer's protocol. GeneChip^®^ Human Gene 1.0ST Arrays and GeneChip^®^ Mouse Gene 2.0 ST Arrays (Affymetrix) were used to determine the expression profiles. Total RNAs were processed using the Ambion WT expression kit (Life Technologies) and the Affymetrix WT Terminal & Labeling kit before being hybridized on Affymetrix arrays (protocol P/N 702808 Rev.6). Upon hybridization, microarrays were washed, stained and scanned according to manufacturer's standard procedures. CEL files containing hybridization raw signal intensities were imported into the Partek GS software for statistical analysis. First, probe intensities were summarized to gene expression signals using Partek default options (GC-content adjustment, RMA background correction, quantile normalization, log2 transformation and summarization by means. R statistical environment was used for hierarchical clustering, principal component analysis and for empirical Bayesian statistics (LIMMA [39], R/Bioconductor). Lists of differentially expressed genes (DEG) were obtained with the eBayes/LIMMA. FDR was calculated with the Benjamini and Hochberg approach [40]. Thresholds were set up for FDR < 0.01 and absolute fold change (abs(FC)) ≥ 2 for human specific microarrays and FDR < 0.01, any FC for mouse specific microarrays. The DAVID^®^ database [41] and the Ingenuity Pathway Analysis (IPA; Ingenuity Systems) were used for data mining. REVIGO was used for summarization of altered gene ontology (GO) terms [42]. Venn diagram analysis was performed with the SUMO software (http://angiogenesis.dkfz.de/oncoexpress/software/). IPA was used for the identification of the DEGs with specific location: cell membrane, extracellular matrix. Microarray data are available in the ArrayExpress database (https://www.ebi.ac.uk/arrayexpress/) under accession numbers E-MTAB-3948 and E-MTAB-3949.

Putative protein-protein interaction analysis was performed for proteins located at the plasma membrane and extracellular matrix encoded by the DEGs up-regulated in angiogenic tumours (P13 vs.P8, FDR < 0.01, FC > 2) and up-regulated in EC (P13 vs. NB ECs, FDR < 0.01, FC > 1). Mouse genes were mapped to human homologues and unique and unambiguous gene symbols were considered for further analyses. Putative protein-protein interactions (PPI) were identified with the GeneMania server [43] from 11 publicly available interaction repositories: BIND, BIND-TRANSLATION, CORUM, DIP, GRID, HPRD, INTACT, MINT, MPPI, OPHID, as well as expert-curated PPIs from small-scale experiments. The search focused on human proteins and physical interactions, and an automatic weighting of retrieved interactions was applied. Cytoscapev3.2.1 was used for network generation and visualization.

### Western blot

Representative xenografts and *in vitro* cultured spheroids were used for protein extracts (P8, T101 invasive, P3, T16 intermediate, P13 angiogenic, three animals/group). Proteins were extracted in lysis buffer (7M Urea, 2M Thiourea, 2% Amidosulfobetaine-14, 2x phosphatase inhibitors (Phostop Roche) pH 8.5). Protein concentration was determined with 2D Quant (GE Healthcare). The signal was visualized using the ECL™ Advance Western blot detection kit (GE Healthcare), the ImageQuant LAS4010 imaging station and software (GE Healthcare) (list of antibodies in Suppl. Table 3).

### Human-specific VEGF ELISA

ELISA was performed following the manufacturer's instructions (Ab100663, Abcam). VEGF concentration (pg/μg of protein) was assessed for the three phenotypes using representative xenografts: (P8, T101 invasive; T16, P3 and NCH421k intermediate; P13, NCH644 angiogenic. Experiments were performed in triplicates with protein extract from 2 animals each. The results are presented per xenograft phenotype.

## SUPPLEMENTARY MATERIAL AND METHODS


