# Supplementary material for: Molecular crosstalk between tumour and brain parenchyma instructs histopathological features in glioblastoma
Source: Oncotarget. 2016 Mar 25;7(22):31955–71. doi: 10.18632/oncotarget.7454 (PMC5077988; doi:10.18632/oncotarget.7454)
Supplement: Supplementary file 1 [file oncotarget-07-31955-s001.pdf]

Molecular crosstalk between tumour and brain parenchyma instructs histopathological features in glioblastoma

SUPPLEMENTARY MATERIAL AND METHODS

Array comparative genomic hybridization (aCGH)

Each patient GBM was analyzed for copy number aberrations using SurePrint G3 Human 2x400k CGH microarrays (Agilent Technologies) as previously described (1). Briefly, genomic DNA was extracted using the DNAeasy Blood and Tissue Kit (Qiagen). DNA was fragmented (200-500bp) by DNAse1 (rDNAse1, Ambion) and labeled with the BioPrime aCGH Genomic labeling Kit (Invitrogen) with Cy3 and Cy5 dyes (GE Healthcare) following standard Agilent protocols. Female DNA pool (Promega) was used as a reference. The slides were scanned at 3μm resolution (Agilent High-Resolution Microarray scanner), the image data was extracted using Feature Extraction (Agilent Technologies). Aberrations were called using the ADM2 algorithm with a threshold setting of 25, centralization ‘on’ and an aberration filter with a minimal number of probes=5 and a minimal AvgAbsLogRatio=0.45.

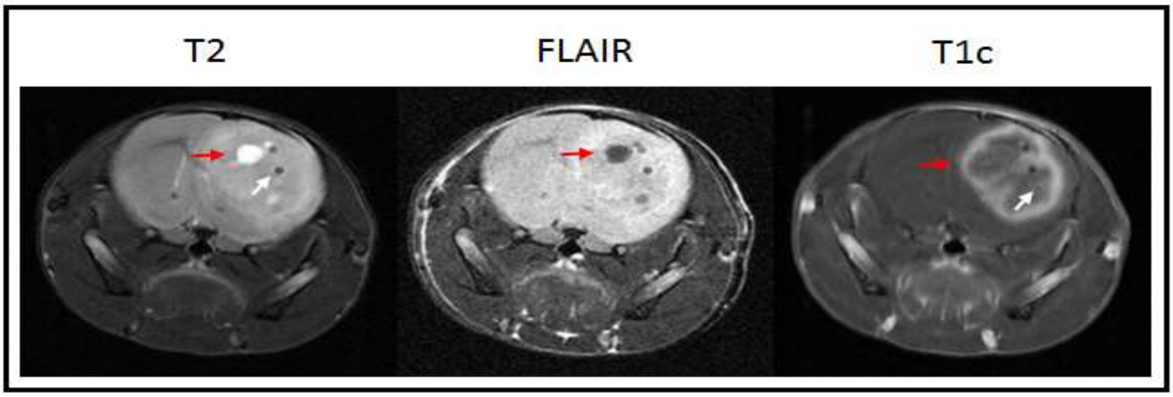

Suppl. Fig 1: MRI anatomical images for a P13 angiogenic tumour in mouse brain (Related to **Fig. 1C**) T2-weighted MRI (left panel) shows a large heterogenous tumour developed in the right hemisphere expanding to the left hemisphere. Dark spots (white arrow) correspond to angiogenic blood vessels and white spots (red arrow) reflect fluid accumulation. The T2-Fluid Attenuated Inversion Recovery (FLAIR) MRI image (center panel) suggests the central white spot in the T2-weighted serie (red arrow) to contain CSF. T1-weighted MRI (right panel) after injection of Gd-based contrast agent reveals a ring of contrast enhancement (red arrow), associated with leaky vasculature, surrounding a central necrotic region (white arrow), in a pattern similar to those observed in clinical Glioblastomas. FLAIR: Fluid Attenuated Inversion Recovery, CSF: CerebroSpinal Fluid, Gd: Gadolinium

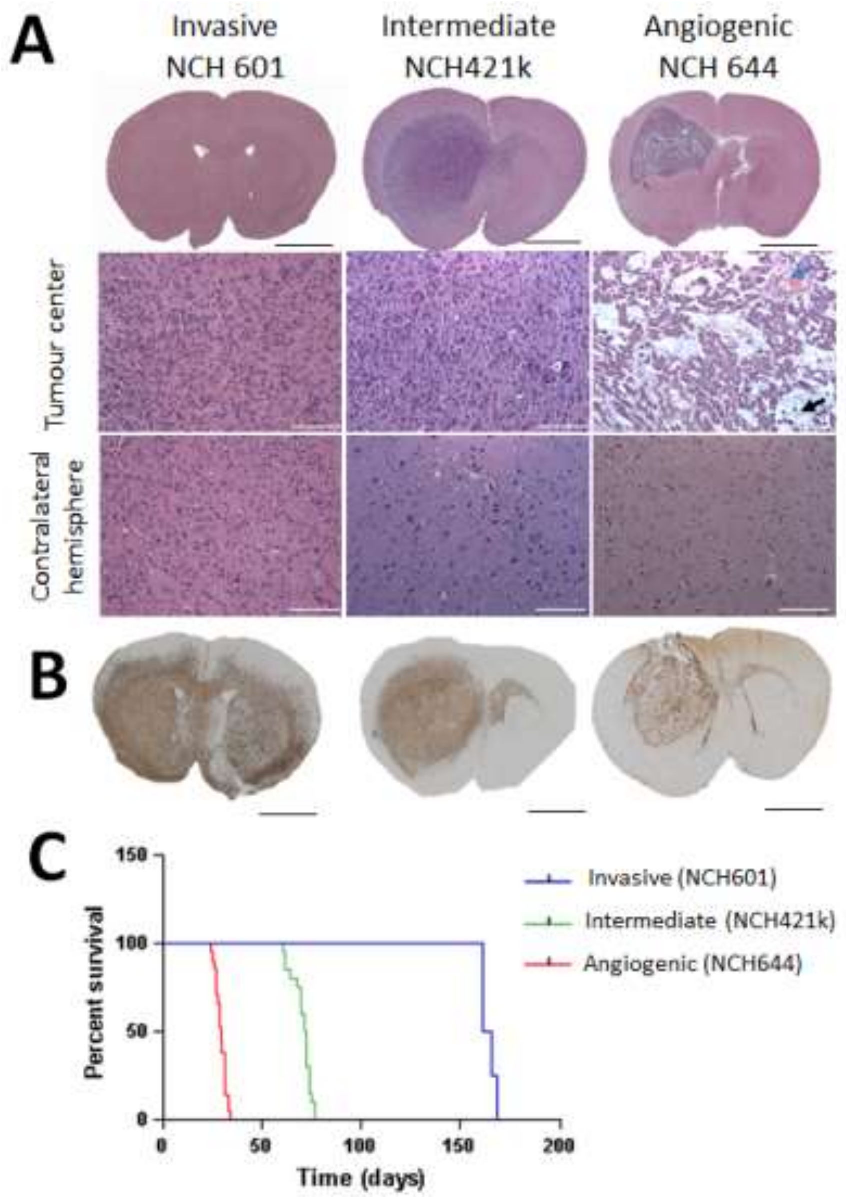

Suppl. Fig. 2: Phenotypes of glioma stem-like cell-derived xenografts (Related to **Fig. 1&2**). Glioma stem-like cultures were implanted intracranially in NOD/SCID mice. The developed tumours were classified into three phenotypes: invasive, intermediate and angiogenic. **A** Hematoxilin/Eosin staining showing tumour center and contralateral hemisphere of xenografts. Examples are shown for NCH601, NCH421k and NCH644. Blue arrow points to vessel with microvascular proliferation, black arrow shows necrosis. White and black scale bars represent respectively 100µm and 1mm. **B** Human-specific nestin (NCH601, NCH421k) or vimentin (NCH644) staining was used to detect tumour cells. Scale bars represent 1mm **c** Kaplan-Meier survival curve of stem-like cell line-derived xenografts (NCH421k and NCH644 n=20; NCH601 n=4).

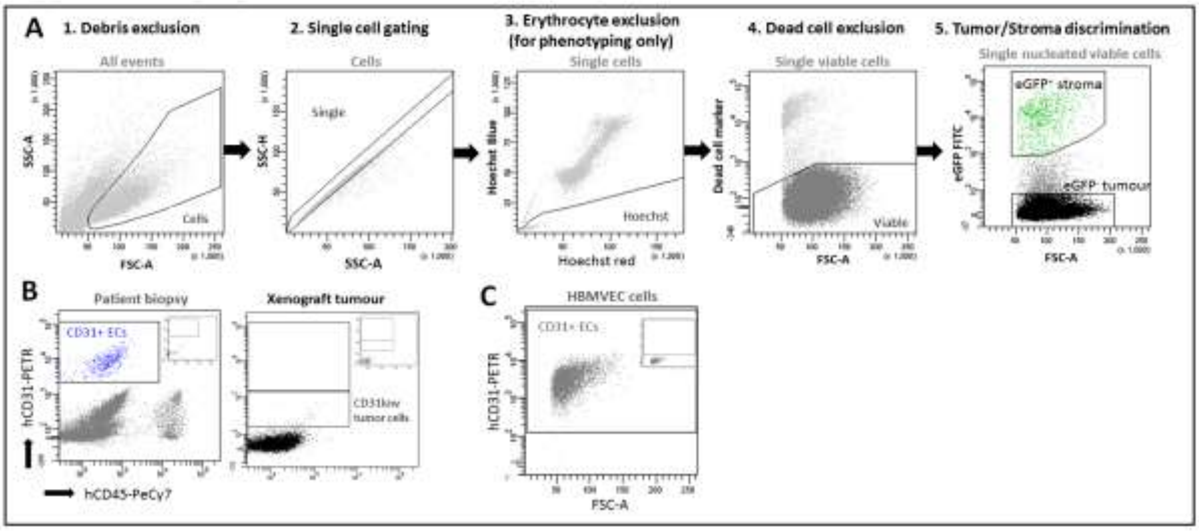

Suppl. Fig. 3: Gating strategy for sorting and multicolor phenotypic analysis (Related to **Fig. 3-6**). **A** Step-by-step gating strategy for FACS analysis is shown for the intracranial P3 xenograft in eGFP^{+} NOD/SCID mice. The same strategy was used for all subsequent experiments using single viable cells, with exclusion of cell debris and erythrocytes. (**1)** Cells were distinguished from debris on the flow cytometric profile based on the Forward Scatter (FSC) and Side Scatter (SSC). (**2)** Cell doublets and aggregates were gated out based on their properties displayed on the SSC area (SSC-A) versus height (SSC-H) dot plot. (**3)** Dead cells were recognized by their strong positivity for the dead cell discrimination marker. (**4)** For multicolor phenotyping, erythrocytes were excluded by applying a 'Hoechst' gate on the 'Hoechst Red'/'Hoechst Blue' dot plot in the linear scale. Hoechst staining was omitted for cell sorting due to increased toxicity (**5)** In xenografts, human tumour cells were recognized as the eGFP negative population compared to the eGFP positive mouse host cells. **B** Comparison of CD31 expression in patient biopsy and tumour cells of a glioblastoma xenograft (T341). CD31+ **C**. CD31 expression in human brain microvascular endothelial cells (HBMVECs). Although CD31 staining was occasionally detected in a limited number of glioma cells (B; CD31 low tumor cells), the expression level was 10-100 times lower compared to CD31positive stromal cells in patient biopsies (B; CD31+ ECs 'blue') and HBMVECs (C).

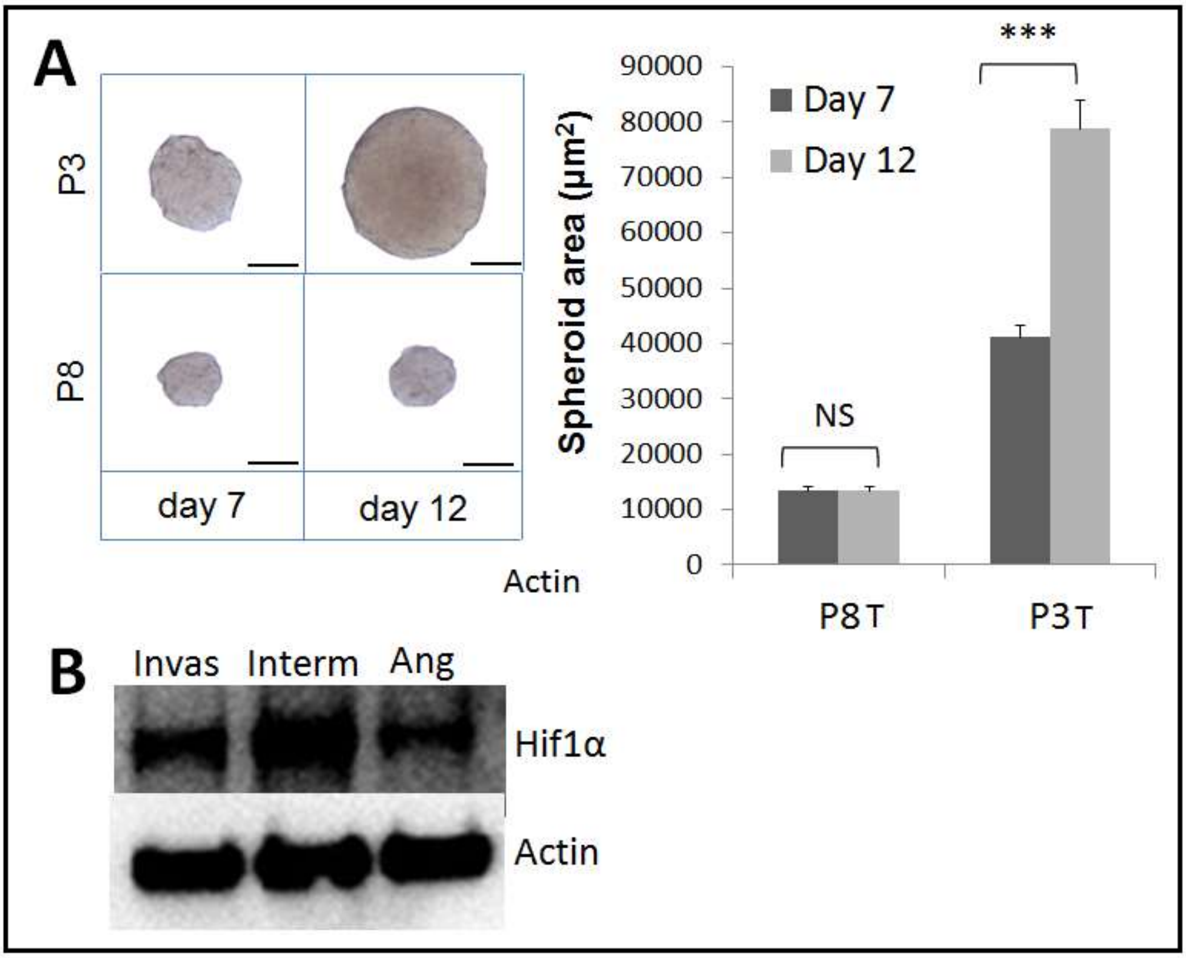

Suppl. Fig. 4: In vitro spheroid cultures. A. (Related to **Fig. 4)** Stroma-free spheroids were assessed for their growth capacities in vitro during 14 days in culture. Size measurements were performed at day 7 and 14. Invasive P8 spheroids hardly proliferated in vitro, whereas P3 intermediate spheroids displayed significant growth over the 14-day-culture. Scale bars represent 100µm; ***pvalue<0.001, n=20 per tumour. **B**. **(**Related to **Fig. 5)** Western Blot analysis showing the HIF1α protein present in organotypic spheroids in vitro of the angiogenic (Ang), intermediate (Interm) and Invasive (Invas) tumours.

Supplementary Figure 5

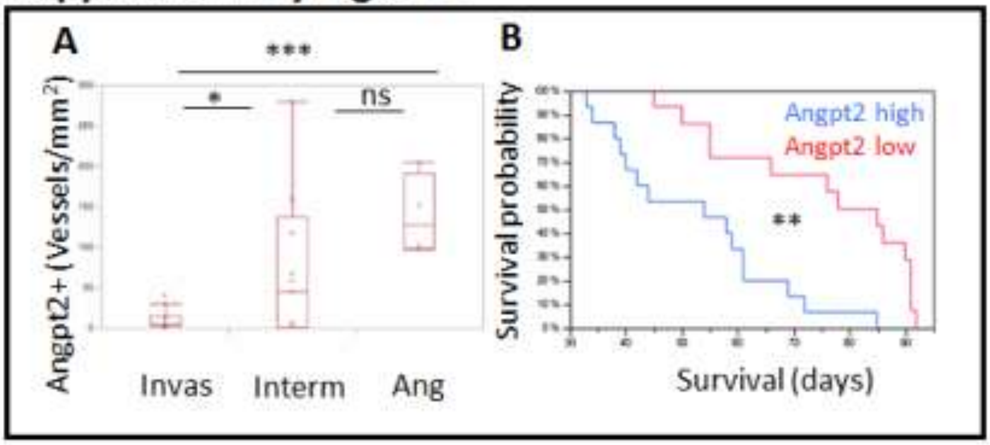

Suppl. Fig. 5: Analysis of Angiopoietin 2 expressing blood vessels (Related to **Fig. 6D). A** Quantification of Angiopoietin 2 (Angpt2) positive vessels confirmed significant differences between intermediate and angiogenic tumours versus invasive tumours. Analysis was performed for invasive (P8, T101, T185, T233, T239, T251), intermediate phenotypes (P3, T16, T238, T341, T434, NCH421k) and angiogenic (P13, NCH644) tumours. **B** Kaplan-Meier survival curve of xenotransplanted mice based on the Angpt2 expression (low/high) in blood vessels; p values were calculated with the Wilcoxon signed-rank test; *pvalue<0.05, **pvalue<0.01, ***pvalue<0.001.

Supplementary Figure 6

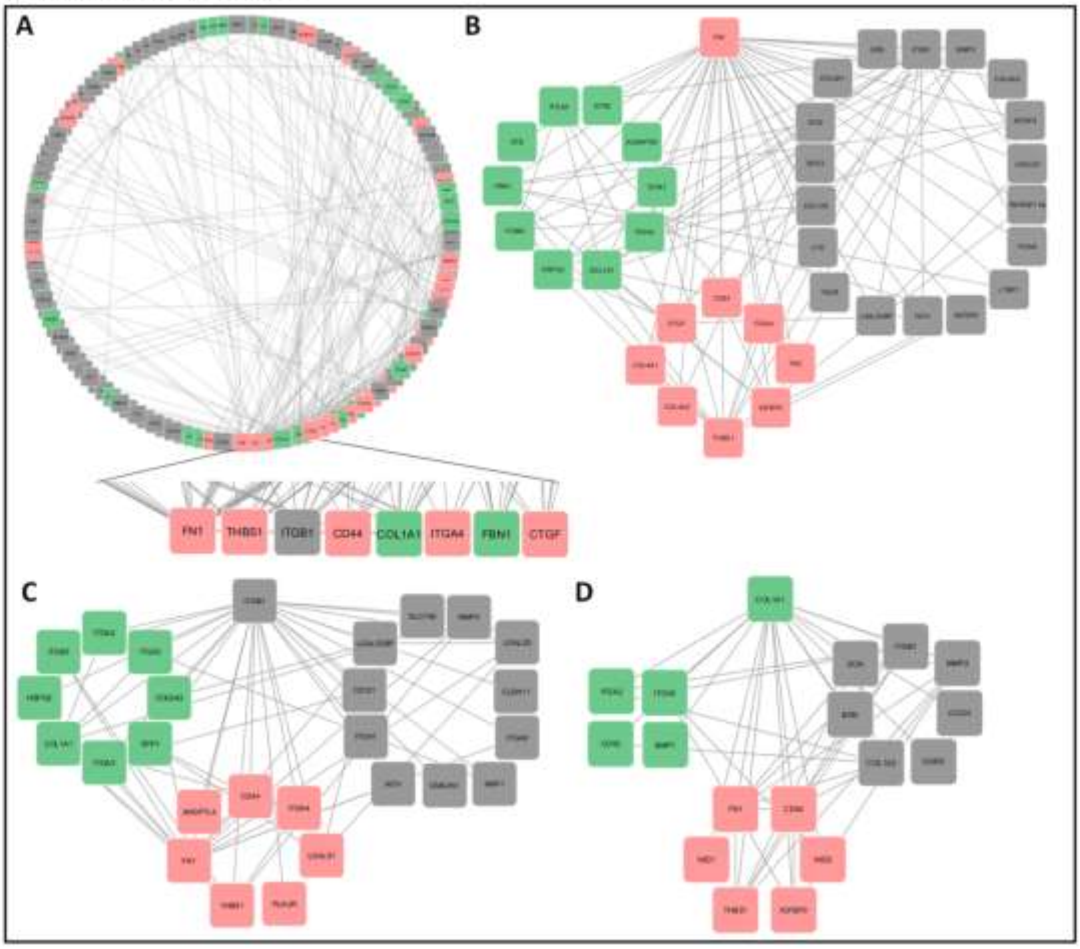

Suppl. Fig. 6: Selected networks for putative protein-to-protein interactions between tumor cells and ECs. A Direct protein-to-protein interactions between tumour and ECs were extracted from the network and displayed on circular layout sorted by degree of interactions (edges). Genes having highest number of interactions are highlighted. **B-D** Selected networks of protein-protein interactions for FN1 (b), ITGB1 (c) and COL1A1 (d) and their direct partners. Selected genes are displayed with their first neighbours grouped by category. Only the direct interactions between molecules upregulated in tumor and ECs are shown.

Suppl. Table 1: Clinical patient data and copy number aberrations of corresponding human glioblastoma biopsies. Genomic aberrations as identified by aCGH are shown for all patient glioblastomas and cell lines usd in the study [++ = amplification (Log2 Ratio>2), + = gain (Log2 Ratio >0.35), - = loss (Log2 Ratio < -0.35), -- = deletion (Log2 Ratio < -1)]. Verhaak glioma subtypes classification (2) for patient biopsies based on the score for Clanc distance is presented when available.

Patient biopsy	Age	Sex	Chromosomal aberrations –Biopsy	Tumor subtypes
T16	52	F	++[EGFR , MDM2], +7q, - [Chr6q, Chr10, Chr11, 13q12-q32.2], --CDKN2A/B	Neural
T101	60	M	++[EGFR, MDM2] +Chr7, -[3q, Chr4, Chr10, Chr11, Chr15], --CDKN2A/B	Mesenchymal
T185	76	F	++EGFR, +Chr7, -10q, --CDKN2A/B	Mesenchymal
T233	43	F	++[EGFR, 2q34] +[1q21.2-24.2, Chr7, Chr19, Chr20], -[9p21.3 -21.1, Chr10], --CDKN2A/B	na
T238	41	M	+Chr7, -[6q, 9p-p21.1,Chr10, Chr13]	na
T239	79	M	+[Chr7, Chr19], -[1p-p35.3, 9p24.2-p23, 9p21.3-p21.1], --CDKN2A/B	na
T251	43	F	++[EGFR, 2q34], +[1q21.2-24.2, Chr7, Chr19, Chr20], -[9p21.3 -21.1, Chr10], --CDKN2A/B	na
T341	75	F	++[PDGFRA, 7p21.1, EGFR, 7q21.1-22.2, 17p12], 1[Chr7, Chr16, Chr19], -Chr10, --CDKN2A/B	na
T434	54	M	++[EGFR, NF1, MDM2], +[7p,20], -[4p, 6q26-qter, 9p, 9q22.32-qter, 10, 14q13-qter]	na
P3	64	M	+ [Chr 7, Chr19, 20q], -[1q42-q43, Chr9, Chr10, 20p] –[PIK3R , CDKN2A/B]	Mesenchymal
P8	64	F	++EGFR, +[Chr7, 8q24], -[6q22-q24, Chr10, 13q13.3-q33.3, 18q21.2-q22.4], --CDKN2A/B	Neural
P13	UN	F	+(Chr7, Chr19, Chr20), -(6q16.2-16.3, Chr10, 17q12), --CDKN2A/B	Neural
Cell Line				
NCH421k	66	M	++[PDGFRA, MYC, CDK4], +[1p31.1-q43, 5q1-q22.2, 16p], -[2q, 3p-q13.31, 8p, 9p, Chr10, 13q]	Proneural
NCH644	67	F	++MYC, +[5q34-qter, EGFR, 8p, 8q-8q22.3, 20q11.23-qter, Chr21, Chr22], -[5q32.2-q34, 12q24.12-q24.32, 13q-q31.1, 18q, Chr19]	Proneural
NCH465	63	M	--[CDKN2C, CDKN2A/B], -10 (complex genome)	na
NCH601	76	M	++MycN, +7,--CDKN2A/B, -[4, 5, 6, 8, 10, 18]	na
NCH660h	74	F	+[5, 7, 12, 14, 16, 17, 21], --[CDKN2A/B], –[2, 6]	na

UN= unknown

Suppl. Table 2: Glioblastoma xenografts generated in NOD/SCID mice. All xenografts used in the study are shown. For the development time, the mean time to sacrifice (days +/- SEM) and the number of mice per group (*n*) are indicated.

**Xenograft**	**Model**	**Phenotype**	**Generations after serial transplantation**	**Number of animals**	**Development time (NOD/Scid mice)**		
					Generation	Survival Days	Number of Mice
T16	Spheroid	Intermediate	6	66	G1	138+/-2	4
					G2	100.8+/-11.1	17
					G3	71.2+/-0.4	5
					G4	76.1+/-7.1	8
					G5	72.8+/-3.6	13
					G6	75.8+/-8.4	19
T101	Spheroid	Invasive	7	44	G1	111.8+/-4.3	10
					G2	117.3+/-3.1	7
					G3	104.9+/-15.1	8
					G4	104.2+/-1.5	4
					G5	101+/-1.5	6
					G6	93	5
					G7	107.7+/-1.5	4
T185	Spheroid	Invasive	3	17	G1	143.3+/-1.2	6
					G2	107+/-2	4
					G3	83+/-5.3	7
T233	Spheroid	Invasive	2	6	G1	152+/-7	3
					G2	107.7+/-4	3
T238	Spheroid	Intermediate	3	10	G1	140+/-2	3
					G2	155+/-8.5	2
					G3	84.4+/-4.9	5
T239	Spheroid	Invasive	1	3	G1	139.3+/-4.6	3
T251	Spheroid	Invasive	3	19	G1	122.5+/-1.7	4
					G2	86+/-4.2	8
					G3	71+/-1.5	6
T341	Spheroid	Intermediate	2	9	G1	58.5+/-0.6	4
					G2	54.6+/-6	5
T434	Spheroid	Intermediate	2	8	G1	61	3
					G2	45	5
P3	Spheroid	Intermediate	6	38	G1	42	3
					G2	48.4+/-1.7	8
					G3	41.3+/-0.5	6

					G4	43.6+/-0.5	5
					G5	39.4+/-0.7	11
					G6	40+/-0.7	5
P8	Spheroid	Invasive	3	31	G1	65.5+/-1	4
					G2	61+/-5.2	18
					G3	52.2+/-0.8	9
P13	Spheroid	Angiogenic	3	31	G1	56	1
					G2	41.9+/-2.4	20
					G3	40.3+/-2.7	10
NCH421k	Neurosph.	Intermediate	1	20	G1	70.4+/-4.8	20
NCH644	Neurosph.	Angiogenic	1	21	G1	29.3+/-2.6	20
NCH465	Neurosph.	Invasive	1	6	G1	133.7+/-12.6	6
NCH601	Neurosph.	Invasive	1	4	G1	165+/-1.7	4
NCH660h	Neurosph.	Invasive	1	8	G1	258.5+/-16.6	8

Suppl. Table 3: List of antibodies used in the study.

Epitope	Conjugate	Species reactivity	Clone	Supplier	Concentration used/test*
CD31	Dy590 (PE-TR)	human	MEM-05	Immunotools	F: 10µl/test
CD31	APC	mouse	390	eBioscience	F: 2.5µl/test
HIF1α	-	human	54/HIF-1α	BD transduction lab 610959	WB:1/1000 = 250 ng/ml
HIF2α	-	human	Ep190b	Novus biological NB100-132	WB:1/2000 =500ng/ml
Actin	-	human/mouse/rat	C4	Milllipore MAB 1501	WB:1/6000
CD31	-	mouse	390	Millipore	IHC:1/200
CD31	Dy590 (PE-TR)	human	MEM-05	Immunotools	10µl/test
CD45	PE-Cy7	human	HI30	Immunotools	5µl/test
MCT4	-	mouse/rat/human	H-90	Santa Cruz Biotechnology	IHC:1/200
Nestin	-	human	10C2	Millipore	IHC:1/200
Vimentin	-	mouse/rat/human	EPR3776	Epitomics	IHC:1/200
Angiopoietin 2	-	mouse/rat/human	PA5-27297	ThermoScientific	IHC: 1/200
Ki67	-	mouse/rat/human	SP6	ThermoScientific	IHC: 1/100
Thrombospondin 1	-	mouse/rat/human	A6.1	Thermo scientific, MA5-13398	IHC: 2ug/ml
Anti-mouse -IgG	HRP	mouse		GE Healthcare LNA931V/AG	WB: 1/10 000
Goat anti-rat IgG	Alexa Fluor 555	rat		Invitrogen	IHC:1/1000
Anti-mouse IgG	HRP	mouse		Dakocytomation	Kit concentration
Anti-rabbit IgG	HRP	rabbit		Dakocytomation	Kit concentration
Anti-Mouse IgG	Biotinylated	horse		Vestor labs, BA-2000	IHC:0.2ug/ml

F = Flow cytometry (test 10^6 cells/100µl); WB = Western Blot; IHC = Immunohistochemistry

Suppl. Table 4: Comparison of differentially expressed genes (DEGs) between angiogenic P13, intermediate P3 and invasive P8 tumour cells. Differentially expressed genes between sorted tumour cells of the angiogenic (P13), intermediate (P3) and invasive (P8) tumour cells were determined with the eBayes (Limma) linear model. Cut-offs were set up for FDR<0.01 and abs(FC)>=2. Common and unique genes were established by the SUMO software Venn diagram analysis (http://angiogenesis.dkfz.de/oncoexpress/software/) (not shown). Gradually increased and decreased genes were extracted as genes common in three DEG comparisons (P13vP8, P13vP3 and P3vP8) for upregulated and downregulated genes respectively. Genes upregulated in angiogenic and intermediate versus invasive tumors were extracted from common upregulated genes between P13vP8 and P3vP8. Genes specific for angiogenic tumors were extracted from common upregulated genes between P13vP8 and P13vP3 comparisons. Genes upregulated in invasive and intermediate tumors versus angiogenic tumors were extracted from common downregulated genes between P13vP3 and P13vP8 comparisons. Genes specific for invasive tumors were extracted from common downregulated genes between P13vP8 and P3vP8. Unique genes specific for each comparison are not shown.

For Table S4, please see the attached Excel file

Suppl. Table 5: DAVID analysis for differentially expressed genes (DEGs) between angiogenic (P13) versus invasive (P8) tumour cells. Differentially expressed genes between sorted tumour cells of the angiogenic (P13) versus invasive (P8) phenotype were determined with the eBayes (Limma) linear model. Cut-offs were set up for FDR<0.01 and abs(FC)>=2. 2672 DEGs were divided into upregulated (FC>=2; 1393 genes) and downregulated genes (FC<=-2; 1279 genes). DEG lists were submitted to the DAVID® database (DAVID 6.7; http://david.abcc.ncifcrf.gov/) for functional enrichment analysis. Main significantly deregulated Gene Ontology (GO) terms, UniProt keywords and features are presented if the enrichment score for the annotation cluster was >2 with terms p value<0.05.

For Table S5, please see the attached Excel file

Suppl. Table 6: Upstream regulator analysis between angiogenic (P13) and invasive (P8) tumour cells. Differentially expressed genes between sorted tumour cells of the angiogenic (P13) versus invasive (P8) phenotype were determined with with the eBayes (Limma) linear model. Cut-offs were set up for FDR<0.01 and abs(FC)>=2. The DEG list was submitted to the Ingenu ty® Pathway Analysis for the analysis of upstream regulators. The regulatory network is considered to be significantly activated if z-score >=2 and inhibited if z-score <=2 (p-value of overlap<0.05).

For Table S6, please see the attached Excel file

Suppl. Table 7: DAVID analysis for differentially expressed genes (DEGs) between endothelial cells of the angiogenic (P13) tumour and normal brain. Differentially expressed genes between sorted $eGFP^{+}CD31^{+}$ endothelial cells in P13 xenografts versus endothelial cells in normal mouse brain were determined with the eBayes (Limma) linear model. Cut-off was set up for FDR<0.01. 635 DEGs were divided into upregulated (447) and downregulated genes (188). Separate DEG lists were submitted to the DAVID® database (DAVID 6.7; http://david.abcc.ncifcrf.gov/) for functional enrichment analysis. Main significantly deregulated Gene Ontology (GO) terms, UniProt keywords and features are presented if the enrichment score for the annotation cluster was >2 with terms pvalue<0.05.

For Table S7, please see the attached Excel file

Suppl. Table 8: Comparison of differentially expressed genes (DEGs) in endothelial cells of angiogenic (P13) tumour, intermediate (P3) tumour and normal mouse brain. Differentially expressed genes between sorted eGFP^{+}CD31^{+} mouse endothelial cells in P13 and P3 xenografts versus endothelial cells in normal mouse brain were determined with the eBayes (Limma) linear model. Cut-off was set up for FDR<0.01. DEGs were divided into upregulated and downregulated genes. Common and unique genes were established by the SUMO software Venn diagram analysis (http://angiogenesis.dkfz.de/oncoexpress/software/).

For Table S8, please see the attached Excel file

Suppl. Table 9: Upstream regulator analysis between endothelial cells of angiogenic tumour (P13) and normal brain. Differentially expressed genes between sorted endothelial cells of the angiogenic (P13) tumour and normal brain were determined with the eBayes (Limma) linear model. Cut-off was set up for FDR<0.01. The DEG list was submitted to the Ingenuity® Pathway Analysis for the analysis of upstream regulators. The regulatory network is considered to be significantly activated in tumour endothelial cells if z-score >=2 and inhibited if z-score <=2 (pvalue<0.05).

Upstream Regulator	Activation z-score	p-value of overlap
**Activated**		
FOXO1	3.94	4.68E-13
FOXM1	3.89	9.41E-18
TNF	3.77	4.15E-10
ERBB2	3.49	1.31E-31
TGFB1	3.29	2.63E-32
S100A6	2.81	1.63E-06
ERK	2.80	3.16E-03
CTNNB1	2.59	1.63E-06
TRAF2	2.44	1.58E-06
BRD4	2.44	7.79E-03
NFkB (complex)	2.39	4.56E-02
Mek	2.30	3.78E-03
CD24	2.24	2.11E-03
P38 MAPK	2.23	1.60E-05
HIF1A	2.21	6.83E-04
EGFR	2.21	5.69E-04
CSF2	2.14	1.95E-03
RLIM	2.00	1.78E-04
PAF1	2.00	3.55E-02
**Inhibited**		
TP53	-5.55	3.22E-17
NUPR1	-4.96	1.82E-07
estrogen receptor	-3.77	8.43E-05
SPDEF	-3.32	2.77E-07
KDM5B	-3.21	3.99E-08
MGEA5	-3.13	6.46E-05
WISP2	-2.43	9.99E-04
miR-34a-5p (and other miRNAs w/seed GGCAGUG)	-2.42	3.95E-04
CDKN1A	-2.27	1.91E-09

ATF3	-2.24	2.85E-06
UXT	-2.23	3.14E-06
miR-483-3p (miRNAs w/seed CACUCCU)	-2.23	1.35E-02
KDM5A	-2.00	5.23E-05
TAB1	-2.00	5.00E-03
DNMT3B	-2.00	1.40E-01

Suppl. Table 10: Comparison of differentially expressed genes in tumour and ECs of the angiogenic phenotype. DEG lists of tumour cells (P13 v. P8 tumour cells, FDR<0.01, abs(FC)>=2) and stromal ECs (P13 v. NB, FDR<0.01, any FC) were compared. Prior to analysis mouse gene IDs were mapped to human gene IDs and unique symbols were extracted. Both DEG lists were split into up and down-regulated genes. Common genes were extracted from Venn diagrams (http://sablab.net). 75 upregulated and 38 downregulated genes were common, 39 genes had an inverse expression levels (not shown).

Suppl. Table 11: Integrative analysis of protein-protein interactions between tumour and stromal ECs. Cell membrane and extracellular matrix-associated genes upregulated in tumour cells (P13 v. P8 tumour cells, FDR<0.01, FC>=2) and ECs (P13 v. NB, FDR<0.01, FC>1) were compared. Prior to analysis mouse gene IDs were mapped to human gene IDs and unique symbols were extracted. Only direct protein-protein interactions between tumour and EC molecules are shown in the table (tumor specific genes in grey, EC specific genes in green, tumor and EC common genes in red).

For Table S11, please see the attached Excel file

Table 12: Comparison with study by Dieterich et al. (3). DEGs of mouse ECs from angiogenic tumours (P13 v. NB, FDR<0.01, any FC; 599 genes) were compared to previously published list of DEGs in human stroma (Glioblastoma patients v. NB; FDR<0.05, any FC; 95 genes) (3) . Prior to analysis mouse gene IDs were mapped to human gene IDs and unique symbols were extracted. Both DEG lists were split into up and down-regulated genes. Common genes were extracted from Venn diagrams (http://sablab.net).

Common genes deregulated between mouse and human ECs (Glioblastoma v. NB)	
**Common upregulated**	**Common downregulated**
*ANGPT2**	*SLC6A1*
*CD93**	
COL4A1	
COL4A2	
COL6A3	
FN1	
HSPG2	
IGFBP4	
ITGA5	
LAMA4	
LAMB1	
MCAM	
*MMRN1**	
NID1	
NID2	
*NR5A2**	
PXDN	
SERPINH1	
WEE1	

*Genes proposed to be unique to ECs and not present in pericytes by Dieterich et al.

REFERENCES

1. Golebiewska A, Bougnaud S, Stieber D, Brons NH, Vallar L, Hertel F, Klink B, Schrock E, Bjerkvig R and Niclou SP. Side population in human glioblastoma is non-tumorigenic and characterizes brain endothelial cells. Brain : a journal of neurology. 2013; 136(Pt 5):1462-1475.

2. Verhaak RG, Hoadley KA, Purdom E, Wang V, Qi Y, Wilkerson MD, Miller CR, Ding L, Golub T, Mesirov JP, Alexe G, Lawrence M, O'Kelly M, Tamayo P, Weir BA, Gabriel S, et al. Integrated genomic analysis identifies clinically relevant subtypes of glioblastoma characterized by abnormalities in PDGFRA, IDH1, EGFR, and NF1. Cancer cell. 2010; 17(1):98-110.

3. Dieterich LC, Mellberg S, Langenkamp E, Zhang L, Zieba A, Salomaki H, Teichert M, Huang H, Edqvist PH, Kraus T, Augustin HG, Olofsson T, Larsson E, Soderberg O, Molema G, Ponten F, et al. Transcriptional profiling of human glioblastoma vessels indicates a key role of VEGF-A and TGFbeta2 in vascular abnormalization. The Journal of pathology. 2012; 228(3):378-390.
